# New Putative Chloroplast Vesicle Transport Components and Cargo Proteins Revealed Using a Bioinformatics Approach: An *Arabidopsis* Model

**DOI:** 10.1371/journal.pone.0059898

**Published:** 2013-04-01

**Authors:** Nadir Zaman Khan, Emelie Lindquist, Henrik Aronsson

**Affiliations:** Department of Biological and Environmental Sciences, University of Gothenburg, Gothenburg, Sweden; Arizona State University, United States of America

## Abstract

Proteins and lipids are known to be transported to targeted cytosolic compartments in vesicles. A similar system in chloroplasts is suggested to transfer lipids from the inner envelope to the thylakoids. However, little is known about both possible cargo proteins and the proteins required to build a functional vesicle transport system in chloroplasts. A few components have been suggested, but only one (CPSAR1) has a verified location in chloroplast vesicles. This protein is localized in the donor membrane (envelope) and vesicles, but not in the target membrane (thylakoids) suggesting it plays a similar role to a cytosolic homologue, Sar1, in the secretory pathway. Thus, we hypothesized that there may be more similarities, in addition to lipid transport, between the vesicle transport systems in the cytosol and chloroplast, i.e. similar vesicle transport components, possible cargo proteins and receptors. Therefore, using a bioinformatics approach we searched for putative chloroplast components in the model plant *Arabidopsis thaliana*, corresponding mainly to components of the cytosolic vesicle transport system that may act in coordination with previously proposed COPII chloroplast homologues. We found several additional possible components, supporting the notion of a fully functional vesicle transport system in chloroplasts. Moreover, we found motifs in thylakoid-located proteins similar to those of COPII vesicle cargo proteins, supporting the hypothesis that chloroplast vesicles may transport thylakoid proteins from the envelope to the thylakoid membrane. Several putative cargo proteins are involved in photosynthesis, thus we propose the existence of a novel thylakoid protein pathway that is important for construction and maintenance of the photosynthetic machinery.

## Introduction

Chloroplasts in plants contain three distinct membrane systems and three compartments with soluble contents. The outer and inner envelope membranes surround the chloroplast, while the thylakoid membrane inside the chloroplast forms the stroma and grana lamellae housing the photosynthesis machinery. The compartments with soluble contents are the intermembrane space between the inner and outer envelope membranes, the stroma between the inner envelope membrane and the thylakoid membrane, and the lumen enclosed by the thylakoids. Chloroplast-localized proteins are derived from both the chloroplast and nuclear genomes, but the vast majority (∼95%) are nucleus-encoded, targeted to the chloroplast and imported across the envelope membranes [1]. This import is facilitated by TOC/TIC translocons at the outer/inner envelope membranes of chloroplasts [2]. On reaching the stroma, proteins targeted to the thylakoid membrane are further distributed by one of four pathways identified to date: the Signal Recognition Particle (SRP) pathway, Secretory (Sec) pathway, Twin Arginine Translocation (Tat) pathway, or spontaneous pathway. Each pathway has a specific combination of energy requirements and substrates. Lumen proteins are transported across the thylakoid membrane via the Sec or Tat pathway, whereas integral thylakoid membrane proteins are transported via the SRP or spontaneous [3]–[5].

Thylakoid membrane lipids are produced at the envelope and must be transferred to the thylakoid membrane [6], [7]. Analyses by several authors indicate that they are transported in vesicles [8]–[10], but it is not known whether vesicles also transport proteins to the thylakoid membrane. The first evidence of vesicle transport inside the chloroplasts was observed in *Pisum sativum* (pea), *Glycine max* (soybean), *Spinacia oleracea* (spinach) and *Nicotiana tabacum* (tobacco) at low temperatures [11], and it has been suggested that many of the factors required for vesicle formation and fusion in the chloroplast are similar to those of the well-characterized vesicle transport system in the cytosol [12]. The latter involves the production of vesicles coated by clathrin and coat proteins I and II (COPI and COPII) [13], [14]. In the cytosolic vesicle transport system proteins are sorted into vesicles that are released from a donor compartment and transferred to an acceptor compartment by fusion with its membrane. COPII vesicles are coated vesicles that deliver cargo from the endoplasmatic reticulum (ER) to the Golgi. COPII vesicle transport involves the following phases: initiation, coat assembly, budding, tethering and finally fusion. The first phase occurs at the donor membrane through activation of Sar1 by a guanine nucleotide exchange factor (GEF), Sec12, which induces a conformational change in Sar1 resulting in its membrane attachment via exposure of a hydrophobic tail [15]. Phase two starts with recruitment of the coat protein complexes Sec23–Sec24 and Sec13–Sec31. Sec23 acts as a GTPase-activating protein (GAP) for Sar1, whereas Sec24 is responsible for binding to membrane-spanning proteins, such as receptors for soluble and transmembrane cargos [16], [17]. The outermost coating of the vesicles consists of the Sec13–Sec31 complex, whose function is not completely understood, although it has been suggested to help in membrane curvature [18].

Several proteins have been previously designated as putative components of the chloroplast vesicle transport system that are mainly homologues of the *Saccharomyces cerevisiae* (yeast) cytosolic COPII vesicle transport components Sar1, Sec13, Sec23, Sec24, Sec31 [19]. The chloroplast homologue to Sar1 was named CPSAR1 (where CP = chloroplast localized) and was further characterized and shown to be involved in thylakoid biogenesis [9]. CPSAR1 is found in vesicles, stroma and the donor membrane, but not in the target membrane, supporting the possibility that it has a similar function to Sar1 [9]. The other putatively chloroplast-localized proteins, not yet characterized, have suggested involvement in vesicle budding at the donor membrane (envelope) in a similar fashion to counterparts in cytosolic vesicle transport [19]. However, no cargo proteins or proteins mediating cargo transport have been identified in chloroplasts.

Cargo proteins are attracted to the vesicle before budding occurs. Two types of cargo proteins are predicted: transmembrane and soluble cargo proteins. Transmembrane cargo proteins are simply attached to the vesicle membrane by interacting directly with the coat via specific diacidic, dihydrophobic or di/mono basic amino acid motifs located on their cytoplasmic sides. In contrast, the link between the coat proteins of the vesicle and the soluble cargo proteins is indirect and mediated by a cargo protein receptor. Cargo protein receptors could interact with coat proteins of the vesicles using either a dihydrophobic or a dilysine/basic amino acid motif, and recognize soluble cargos that have an ILV motif [20]–[22].

Although CPSAR1 is the only protein that has been associated with chloroplast vesicles *per se* [9] to date, several other proteins have suggested roles in thylakoid biogenesis related to vesicle transport in chloroplasts [23]–[26]. One of them, VIPP1 (vesicle-inducing protein in plastids 1), has been proposed to interact with the chloroplast protein import apparatus for further transport of nucleus-encoded proteins to the thylakoids [27]. Interestingly, it was speculated recently that the light-harvesting chlorophyll a/b-binding protein B1 (LHCb1), which is important for photosynthesis, might be targeted to thylakoids via vesicle transport [25] during an early developmental stage. This opens the possibility that chloroplast vesicles may transport proteins in addition to lipids, although no cargo proteins have been confirmed in them. These findings, in combination with the presence of vesicles in chloroplasts similar to those of the cytosolic pathway, and the discovery and characterization of CPSAR1, imply that the chloroplast should contain more counterparts of cytosolic vesicle transport system proteins with roles in fusion e.g. Rab GTPases, SNAREs and tethering factors.

Rab GTPases are a large group of small GTPases. They are involved in several processes in vesicular trafficking in eukaryotic cells, from uncoating of vesicles, to tethering and fusion [28]. Like other Ras superfamily members, Rab GTPases undergo a functional cycle. When the Rab proteins are in the soluble or inactive state they are bound to a GDP dissociation inhibitor (GDI). Their dissociation from the GDI is catalyzed by a GDI displacement factor (GDF) that sequesters the Rab proteins and ensures their retention in the membrane by restraining action of the GDI. When bound to the membrane Rabs are in the active state with GTP bound with the help of a GEF, and can be inactivated via hydrolysis of the GTP by a GAP [29].

Before fusion, tethering factors help in pairing vesicles with the donor membrane for proper recognition. Finally, interactions between soluble N-ethylmaleimide-sensitive factor attachment protein receptors (SNAREs), located on both the vesicles (v-SNAREs) and the target membrane (t-SNAREs), are required for fusion of the vesicles and delivery of the cargo protein at the target membrane [30]–[32]. The v- and t-SNAREs form a *trans*-complex, possibly assisted by reticulon proteins. The precise role of reticulons is not known, but evidence provided by several authors [33]–[35] suggests they are involved in vesicle trafficking, particularly during fusion.

Here, we propose that if a COPII-related vesicle transport system exists in chloroplasts there should be evidence for additional vesicle transport components and cargo proteins similar to those of the cytosolic vesicle transport system. Therefore, we have sought for such proteins, and propose a model based on putative chloroplast counterparts of the cytosolic vesicle transport system. According to our model, cargo proteins should exist and be selected at the chloroplast envelope membrane (donor membrane). Then, as the vesicles bud from the donor membrane they should shed their coats and travel through the stroma, with proteins attached to their surface required for anchorage when they reach the thylakoid membrane (target membrane). This implies that chloroplast transmembrane cargo proteins as well as cargo receptors should have appropriate motifs directed into the stroma that are exposed for interaction with the coat proteins of the vesicle. Thus, we also propose the existence of components involved in fusion events e.g. tethering factors, SNAREs, Rabs and reticulons, as in the cytosolic vesicle transport system. Accordingly, as described below, we identified putative components required for each phase of chloroplast vesicle transport (not only the budding phase) using a bioinformatics approach. Furthermore, we identified putative cargo proteins and receptors, and hence propose a possible fifth thylakoid protein targeting pathway. Thus, a chloroplast vesicle transport system may transfer not only lipids to the thylakoid membrane, but also cargo proteins. In addition, since several identified cargo proteins are involved in photosynthesis, construction and maintenance of the photosynthetic machinery may be dependent on vesicle transport.

## Methods

**Figure 1:**
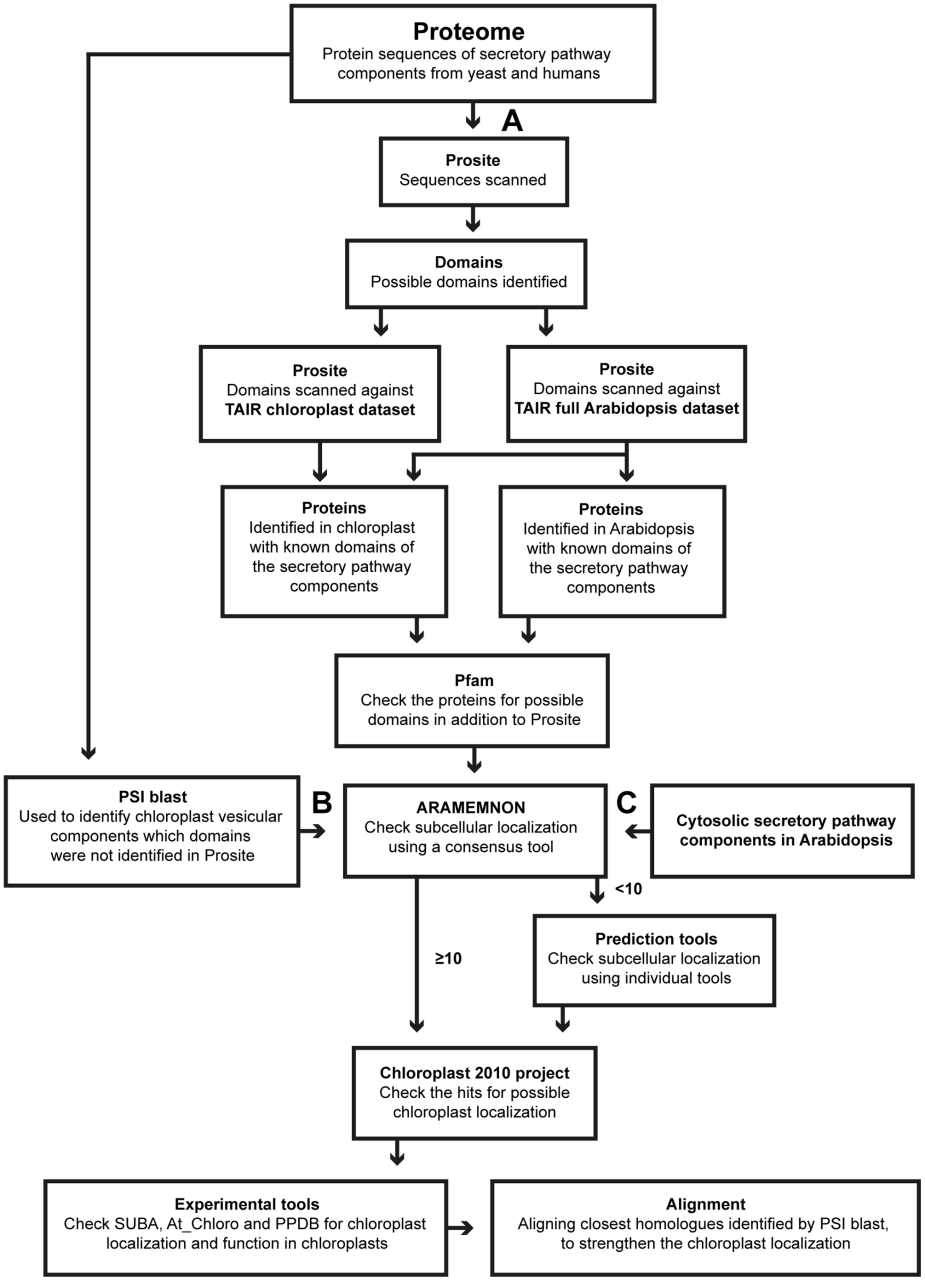
Identification of putative chloroplast vesicle transport components in Arabidopsis. Schematic work flow of the bioinformatics methods used to find putative vesicle transport proteins in chloroplasts. **A**, characteristic domains for cytosolic vesicle transport proteins identified using Prosite; **B,** Possible chloroplast vesicle components identified using yeast protein sequences as queries in PSI blast search of the Arabidopsis proteome. **C**, Arabidopsis cytosolic secretory pathway components used to search for putative chloroplast homologues. ≥ consensus score above or equal to 10;<consensus score below 10.

**Figure 2:**
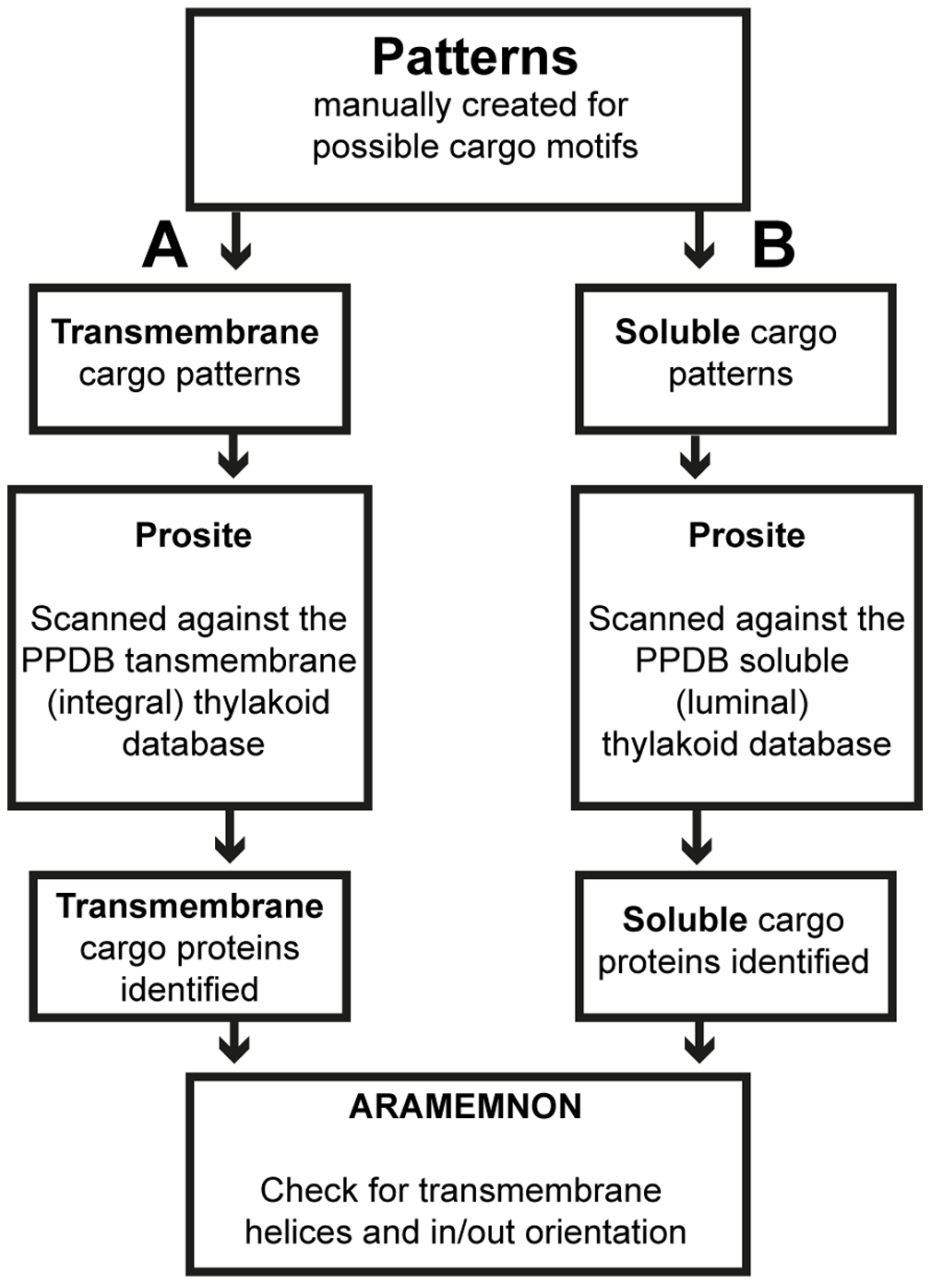
Identification of putative cargo proteins in Arabidopsis chloroplasts. Schematic work flow of the bioinformatics methods used. The manual creation of the patterns was based on previous findings of motifs present in cargo proteins of the cytosolic vesicle transport system. **A**, transmemembrane cargo proteins’ patterns; **B**, soluble cargo proteins’ patterns.

The workflow is schematically depicted in Figures 1 and 2, and described in detail below.

### Protein Datasets Used

Protein sequences corresponding to yeast and *Homo sapiens* (human) proteins involved in initiation, assembly and budding of COPII-related vesicles at the donor membrane (ER), and both fusion and cargo delivery at the acceptor membranes (Golgi, plasma membrane) in the cytosolic vesicle transport system were retrieved from the literature and used as starting material (Figure 1) [32], [36]–[42]. COPI and clathrin-related proteins involved in vesicle initiation, assembly and budding were not included since our aim was to resolve a possible COPII-related vesicle transport system in chloroplasts based on previous notions of COPII-related proteins at this stage [19].

The protein sequence datasets for the full Arabidopsis thaliana proteome (ftp://ftp.arabidopsis.org/home/tair/Proteins/TAIR10_protein_lists/) and Arabidopsis chloroplast proteome (GO: 0009507) were retrieved from TAIR version 10 (Figure 1A). Similarly, an Arabidopsis thylakoid transmembrane and soluble protein datasets was retrieved from the Plant Proteome DataBase (PPDB, http://ppdb.tc.cornell.edu/dbsearch/subproteome.aspx) (Figure 2) [43].

### Identification of Domains, Patterns and Amino Acid Motifs from Cytosolic Vesicle Transport Proteins

Prosite release 20.0 (http://prosite.expasy.org) [44] and Pfam 26.0 (http://pfam.sanger.ac.uk) [45] search tools were used to identify domains, patterns or motifs in our COPII-related protein dataset that may be important for specific functions in cytosolic vesicle transport (Figure 1A). Protein families that have too divergent sequences for identification through these patterns or motifs could still be found using Prosite, since it applies a technique based on weight matrices known as profiling [46]. First, regions of interest assigned by specific entries in Prosite from secretory pathway proteins were identified. After identifying the entries both datasets from TAIR were uploaded and searched in Prosite, to find similar regions first in Arabidopsis and more specifically in Arabidopsis chloroplast-localized proteins. In addition to Prosite the identified proteins in Arabidopsis were scanned in Pfam as well (Figure 1A). The entries are noted in brackets as PS (Prosite) and PF (Pfam).

For proteins lacking a relevant domain according to Prosite we performed a PSI-blast (Position-Specific Iterated BLAST) search at NCBI (http://blast.ncbi.nlm.nih.gov/Blast.cgi) using the NCBI Protein Reference Sequences database and the organism Arabidopsis (taxid:3702) (Figure 1B).

If no defined properties were identified using Prosite and PSI-blast searches, we instead searched in the previous literature regarding cytosolic vesicle transport pathway homologues in Arabidopsis, which were then tested for chloroplast localization (Figure 1C).

### Selection of Cargo Proteins and Receptors

To identify possible cargo proteins spanning the membrane with a helix, diacidic [DE]X[DE] and dihydrophobic [FY](2) motifs were manually created according to rules retrieved from and described in Prosite (Figure 2A). These motifs act as signals and should theoretically be located on the C-terminal side of the cargo protein after the transmembrane helix, and the C-terminal should be directed towards the cytosol [22], or towards the stroma in chloroplasts. A dibasic signal [RK]X[RK], or in some cases a monobasic signal, may be indicative of transmembrane proteins, so this pattern was created for the N-terminal side proximal to the transmembrane helix (Figure 2A) [20]. In chloroplast-localized proteins these signals should be located downstream of the transit peptide.

For soluble cargo proteins lacking a membrane-spanning domain, an ILV (IX(2)LX(9)V) pattern was created that could be present anywhere in the protein [21], except the transit peptide (Figure 2B). For receptors of soluble cargo proteins, two amino acid motifs were mostly required: a dihydrophobic and a dilysine (KX(0,1)KX(2)) or di/mono basic motif. In addition, to predict coiled-coil domains known to be present in cargo receptor proteins we used EMBnet (http://www.ch.embnet.org/software/COILS_form.html).

All the patterns created were scanned using Prosite and patterns found were run against the thylakoid PPDB dataset in Prosite (Figure 2).

### Subcellular Localization of Identified Proteins

The ARAMEMNON release 7 database of plant proteins (http://aramemnon.uni-koeln.de) was used to retrieve information on protein descriptions, predict transmembrane spanning regions and find soluble proteins (Figures 1 and 2) [47]. In addition, ARAMEMNON was used to predict subcellular localizations of proteins based on results collected from the following 17 prediction tools included in the ARAMEMNON: BaCelLo [48], ChloroP_v1.1 [49], iPSort [50], Mitopred [51], Mitoprot_v2 [52], MultiLoc [53], PA-SUB_v2.5 [54], PCLR_v0.9 [55], PProwler_v1.1 [56], PrediSi [57], Predotar_v1 [58], PredSL [59], SignalP_HMM_v3 [60], [61], SignalP_NN_v3 [62], SLP-Local [63], TargetP_v1 [62], [64] and WoLF PSort [65]. Using these tools, a Bayesian consensus (SigConsens) score was obtained from ARAMEMNON for each protein with patterns of interest [66]. A score ≥10 was considered a reliable consensus, providing a strong prediction of subcellular location. The prediction tools were then applied individually to proteins with consensus scores <10 to verify possible chloroplast localization. In these specific cases the additional prediction tools: AdaBOOST [67], MultiP [68], Plant-mPloc [69]–[71], SLPFA [72], and YLoc [73], [74] were included to strengthen the achieved data (Figure 1).

Proteins indicated by ARAMEMNON to have an unknown function were confirmed to have transcripts (cloned cDNA or cognate EST) using TAIR (http://www.arabidopsis.org).

The AT_CHLORO database (http://www.grenoble.prabi.fr/at_chloro) was used to find information on experimental support for the proteins’ localization in different sub-fractions of the chloroplast (Figure 1) [75]. As a complement, SUBA version 2.21 (http://suba.plantenergy.uwa.edu.au/), a database of subcellular locations of proteins supported by experimental data in Arabidopsis, was used to obtain additional information about the experimental methods used (Figure 1) [76]. Finally, a list of proteins predicted to be chloroplast localized from the Chloroplast 2010 project (http://bioinfo.bch.msu.edu/2010_LIMS) was used to further confirm our localization results (Figure 1) [77].

### Alignments to Identify Transit Peptides

For Arabidopsis proteins with unclear chloroplast localization, due either to weak prediction in our analysis or conflicting indications in previous publications about their localization we performed multiple alignments (Figure 1). We included homologous proteins of yeast, humans and Arabidopsis and used Clustal Omega 1.1.0 (http://www.ebi.ac.uk/Tools/msa/clustalo/) [78], [79]. with editing using Box Shade 3.21 (http://www.ch.embnet.org/software/BOX_form.html), to indicate the presence of a transit peptide to support chloroplast localization of the specific protein.

The closest protein homologues of the putative chloroplast-localized proteins used in our multiple alignments were identified by applying a PSI Blast search at NCBI (http://blast.ncbi.nlm.nih.gov/Blast.cgi) using the NCBI Protein Reference Sequences database and the organisms yeast (taxid:4932) and humans (taxid: 9606).

## Results

### Initiation and Budding

Cytosolic vesicle budding is initiated by the recruitment of Sar1 to the donor membrane following its activation to a GTP-bound state from the inactive GDP-bound state by a GEF. The N-terminus of Sec12 in yeast, acting as the GEF of Sar1, contains two WD regions and appears in the cytosol, whereas the C-terminus contains a transmembrane domain and a glycosylation site located in the ER lumen [15]. We identified no clear domains or motifs for Sec 12 in the yeast and human protein dataset using Prosite. However, a PSI blast search identified three homologues to Sec12 in the complete Arabidopsis proteome dataset: AtSec12/At2g01470, At5g50550 (unknown function) and AtPHF1/At3g52190, all possessing WD regions proximal to the N-terminus (PS50294, PS50082) and a transmembrane region in the C-terminus (ARAMEMNON), as well as glycosylation sites (PS00001). Interestingly, the glycosylation sites of the Arabidopsis homologues were positioned in the N-terminus, as opposed to the yeast Sec12. Also, the orientation of AtSec12 and AtPHF1 differed, i.e. the N-terminus was predicted to be directed into the lumen of the ER (AtSec12) or the stroma (AtPHF1), whereas in yeast Sec12 and At5g50550 the N-terminus is directed into the cytosol (ARAMEMNON). According to the localization tools TargetP, MultiLoc, SLPFA and PCLR, AtPHF1 could be located in chloroplasts (Table 1), and in combination with its similarities to yeast Sec12 and AtSec12 it was considered a putative GEF for CPSAR1 in chloroplasts.

**Table 1:** Putative components involved in chloroplast vesicle initiation and cargo protein selection.

Accession No	Name (ARAMEMNON)	SigConsens (ARAMEMNON)			Comment	Localization (TAIR)	Chloro-plast 2010	Chl. loc. (MS/MS)
		Chl.	Mt.	Sec. path.				
**Putative GEF for CPSAR1**								
At3g52190	Phosphate transporter traffic facilitator (AtPHF1)	7.6	0	0.3	Chl., (MultiLoc, PCLR, SLPFA, TargetP)	ER, PM	Chl.	–
**Putative cargo receptors**								
At1g72150	Cell-plate-associated protein (AtPATL1/AtPatellin1)	2.3	0	0	–	Chl., PM, Vacuole	Chl.	[75], [83]
At4g09160	Putative SEC14-like phosphoinositide-binding protein	4.7	0	0	Chl., (Target P)	Chl.	Chl.	[83]
At1g22530	Putative SEC14-like phosphoinositide-binding protein	3.1	0	0	–	Chl., PM	Chl.	[83]
**Putative Sec14**								
At5g63060	Putative SEC14-like phosphoinositide-binding protein	18.1	4.2	0.4	Chl., (ARAMEM-NON)	Chl.	Chl.	[82]
At2g16380	Sec14p-nodulin domain phosphatidyl-inositol transfer protein (AtSFH7)	5.2	0	0	Chl., (ChloroP, PCLR, PredSL)	–	–	–
At3g46450	Putative SEC14-like phosphoinositide-binding protein	6.7	4.2	0	Chl., (ChloroP, PCLR, PProwler, WoLF PSORT)	–	–	–
At2g18180	Sec14p-nodulin domain phosphatidyl-inositol transfer protein (AtSFH10)	5	0	2.7	Chl., (BacelLo, MultiLoc, PredSL, Plant-mPloc, TargetP)	–	Chl.	–

SigConsens, consensus prediction of subcellular localization: Chl., chloroplast; Mt., mitochondria; Sec. path., secretory pathway; Chl. loc., chloroplast localization; MS, mass spectrometry; ER, endoplasmatic reticulum; PM, plasma membrane; Chloroplast 2010, predicted chloroplast localized proteins (http://www.plastid.msu.edu/index.html).

CPSAR1 has been previously characterized [9], [80], [81] and is regarded as a homologue to Sar1 [9], [19]. A PSI blast search showed that CPSAR1 is indeed similar to its yeast counterpart (BLAST score 36, 24% identicality and 48% similarity in amino acid sequence in the first iteration; Table 2), supporting the previous characterization and inference.

**Table 2:** Putative chloroplast-localized *A. thaliana* homologues of yeast COPII proteins.

*S. cerevisiae* COPII component (predicted size in kD)	*A. thaliana* Chloroplast homologues	Blast-Score (% identity/% positive)	E- Value	Coverage (%)	PSI blast iteration	Predicted size (kD)	TargetP reliability class
Sar1 (21.4)	CPSAR1 (At5g18570)*	35.8 (24/48)	6e−05	85	1	75,6	1/5
Sec23 (85.4)	Sec23 (At4g01810)*	116 (22/39)	3e−29	96	1	96	3/5
Sec24 (103.6)	Sec24a (At3g44340)*	306 (27/49)	7e−90	82	1	117.7	3/5
	Sec24b (At4g32640)*	306 (29/49)	4e−90	82	1	116.3	3/5
Sec13 (33.0)	Sec13a (At3g49660)*	68.2 (28/43)	8e−15	78	1	34.8	3/5
	Sec13b (At2g43770)	76.3 (26/41)	2e−17	94	1	37.9	5/5
Sec31 (138.7)	Sec31a (At5g38560)*	N.I	N.I	N.I	5	72.4	1/5
	Sec31b (At2g45000)*	N.I	N.I	N.I	5	73.5	2/5

* Also predicted by [19]; N.I, not identified.

Other proteins involved in vesicle budding in yeast are SEC14 proteins, which contain a CRAL_TRIO domain that plays a role in vesicle transport, possibly during vesicle budding. The CRAL_TRIO domain was identified using Prosite and the TAIR Arabidopsis dataset contained 22 proteins with a single CRAL_TRIO domain (PS50191), designated SEC14 homologues in accordance with their yeast counterpart. All these proteins are designated SEC14-like proteins in ARAMEMNON. One of these proteins, SEC14-like/At5g63060, was found in the TAIR chloroplast dataset by scanning against the Prosite domain. SEC14-like has strong predicted chloroplast localization according to ARAMEMNON, and is present in chloroplasts according to a previous proteomic analysis [82]. The other SEC14-like proteins were scanned using a range of subcellular localization prediction tools and, as shown in Table 1, three were predicted to be chloroplast localized: AtSFH7/At2g16380 (using ChloroP, PCLR and PredSL), SEC14-like/At3g46450 (using ChloroP, PCLR, WoLF PSort and PProwler) and AtSFH10/At2g18180 (using BacelLo, Plant-mPloc, PredSL, TargetP and MultiLoc).

### Coat Assembly

During the budding stage vesicles are coated by coatamer protein pairs consisting of Sec23-Sec24 and Sec13-Sec31 in yeast, which are later uncoated and recycled. The presence of homologues of these coatamer pairs in the chloroplast has been previously predicted, indicating the existence of a COPII-like system in chloroplasts, but this has not been experimentally verified [19]. A PSI blast was performed to support or reject possible homologues of the yeast Sec23-Sec24 and Sec13-Sec31 coatamer pairs. All sequences showed regions of homology in Arabidopsis after one iteration, supporting the earlier predictions, except for the two Sec31 proteins, Sec31a/At5g38560 and Sec31b/At2g45000, which were not identified as homologues even after the fifth iteration (Table 2, Figure S1). Thus, this conflicts the previous prediction of them being Sec31 proteins (Table 2; Andersson and Sandelius, 2004). In support of our notion we did not identify any WD regions in the previously suggested Sec31 homologues, but instead found them to have a protein kinase domain (PF00069, PS50011) for Sec31a, and an Nsp1 domain (PF05064) for Sec31b found in proteins located at the nuclear membrane. However, multiple alignment of the previously suggested Sec13/At3g49660 protein showed clear homology to yeast Sec13, including a conserved WD region (Figure S2). In addition, we identified a second Sec13/At2g43770 protein, possibly residing in chloroplasts, through a PSI blast search (Table 2, Figure S2).

### Cargo Receptors

Receptors for soluble cargo proteins in the cytosolic vesicle transport system are responsible for cargo loading and cargo specificity. In these receptors a Golgi dynamics (GOLD) domain is responsible for selection of cargo proteins [37]. Thus, to identify possible chloroplast cargo receptors we searched for the GOLD domain profile (PS50866) in proteins included in the chloroplast TAIR dataset using Prosite. Three proteins containing the GOLD domain were identified (Table 1): one (Sec14 like/At4g09160) with and two (AtPATL1/At1g72150 and Sec14-like/At1g22530) without a transit peptide according to TargetP. However, all three proteins are located in the chloroplast envelope according to proteomics analysis using SUBA (Figure 1) [75], [83].

Interestingly, the GOLD domain of all three putative cargo receptors is in their C- terminal, whereas in secretory pathway counterparts the GOLD domain is in the N-terminus. Moreover, the cargo receptors of the cytosolic vesicle transport system also have a dilysine motif or several other basic amino acids that interact with the coat proteins in the C-terminal end of the protein. Similarly, the chloroplast cargo receptors possess this motif in their C-terminus, but within the GOLD domain. This suggests that the C-terminal of the proteins could be involved in both cargo protein selection and interaction with the vesicles in chloroplasts.

The putative chloroplast receptors also contained a coiled-coil domain, predicted by EMBnet, towards the N-terminal side, which could interact with other receptors to form tetramers, as in the cytosolic vesicle transport system [84]. In addition, the chloroplast proteins differed from their cytosolic counterparts by not having any transmembrane region but instead an additional domain (the CRAL_TRIO domain; PS50191), involved in vesicle budding and biogenesis. The CRAL_TRIO domain has a hydrophobic lipid binding pocket for phospholipids [85]. After uploading the full Arabidopsis dataset from TAIR (Figure 1), we found five proteins having both a GOLD and a CRAL_TRIO domain in total, including the three putative cargo receptors found in the chloroplast. After aligning all five proteins containing these domains an extra N-terminal stretch was identified in the three putative chloroplast cargo receptors, which could represent a transit peptide responsible for chloroplast localization (Figure S3).

### Cargo Proteins

Cargo proteins known to be transported by the cytosolic vesicle transport system have specific diacidic, dihydrophobic and dibasic signals for recognition and interaction with receptors and membranes. Thus, these signals were used as starting points to manually create patterns using Prosite, then these domains were scanned against the PPDB thylakoid protein dataset (Figure 2), and 32 proteins with cargo signals were identified. Of these, 16 had the diacidic signal and one had the dihydrophobic signal on the C-terminal side, while three had the dibasic motif in their N-terminus (Table 3). In addition, the ILV signal for soluble cargo was found in 12 thylakoid proteins (Table 4).

**Table 3:** Putative thylakoid transmembrane cargo proteins in chloroplasts.

Accession No.	Name (ARAMEMNON)	Putative Motif	TM spans	Orientation
**Putative transmembrane cargos having a diacidic motif at the C-terminus**				
At1g02910	Chloroplast membrane chaperone, required for efficient PSII assembly(AtLPA1)	**E**E**E**, **D**F**D**, **E**I**E**	2	N(In)-C(In)
At1g03160	Dynamin-type GTPase (AtFZL)	**D**I**D**	2	N(In)-C(In)
At1g15820	Light-harvesting chlorophyll a/b-binding protein of minor antennacomplex (AtLHCb6/AtCP24)	**E**P**D**, **D**F**D**	2	N(In)-C(In)
At1g18730	Putative subunit of chloroplastic NAD(P)H dehydrogenase (AtNDF6)	**D**I**E, E**I**E**	1	N(Out)C(In)/N(in)-C(Out)
At1g34000	Putative Lhc protein (AtOHP2)	**D**L**E**	2	N(In)-C(In)
At1g44575	Chloroplast photosystem II 22kDa protein (AtPsbS)	**D**G**E**	4	N(In)-C(In)
At2g18710	Putative secY-type component of plastidic Sec protein translocasesystem (AtSCY1)	**E**L**D**	10	N(In)-C(In)
At2g26500	Putative subunit IV of cytochrome b6f complex	**E**A**E, E**A**E**	1	N(Out)-C(In)
At2g28800	Membrane insertase component of plastidic SRP protein translocasesystem (AtAlb3)	**E**Q**E, E**S**E, DDEEEE**	4	N(In)-C(In)/N(Out)-C(Out)
At2g30570	PsbW-type subunit of photosystem II complex (AtPsbW)	**EEDEE**	2	N(In)-C(In)
At3g08940	Light-harvesting chlorophyll a/b binding protein (AtLHCb4.2)	**D**I**D**	2	N(In)-C(In)
At4g17600	Putative chlorophyll a/b binding protein	**D**V**D, DDDE**	2	N(In)-C(In)
At4g22260	Chloroplast terminal oxidase, product of IMMUTANS gene (AtIM/AtPTOX)	**D**D**D, E**A**E, EDDDTEEE**	2	N(In)-C(In)
At4g31560	Putative membrane protein of unknown function	**D**E**D, E**G**D**	1	N(Out)-C(In)
At5g01530	Light-harvesting chlorophyll a/b binding protein (AtLHCb4.1/AtCP29)	**E**L**D**, **D**S**E**, **D**P**E**	2	N(In)-C(In)
At4g18160	Subunit of dimeric tandem-pore potassium cation channel(AtTPK3/AtKCO6)	**D**I**D**, **E**M**E**, **D**K**D**	5	N(Out)-C(In)
At4g01150	Putative subunit PsaP of photosystem I complex	**EDIE**	2	N(In)-C(In)
**Putative transmembrane cargos having a basic amino acid (s) at the N-terminus**				
At1g06430	Component of Type-B FtsH metalloprotease complex (AtFtsH8)	**KK**	1	N(in)-C(Out)
At1g22700	Tetratricopeptide repeat (TPR)-containing protein (AtPyg7)	**KK**	1	N(in)-C(Out)
At1g51400	Putative subunit PsbT of photosystem II complex	**KK**	1	N(in)-C(Out)
**Putative transmembrane cargos having a dihydrophobic motif at the C-terminus**				
At4g14870	Putative secE-type component of plastidic Sec protein translocasesystem (AtSECe1)	**FF**	1	N(Out)-C(In)

TM, transmembrane; N, N-terminal; C, C-terminal; In, towards the stroma; Out, towards the cytosol.

**Table 4:** Putative thylakoid soluble cargo proteins with an ILV motif.

Accession No.	Name (ARAMEMNON)	Putative motif
At1g64770	Putative component of subcomplex B of chloroplast NDH (AtNDF2/AtNDH45)	**I**DA**L**QIELSCTAG**V**, **I**VS**L**YPVSMATAL**V**
At1g80030	Putative DnaJ-type (I) heat shock system associated protein (AtDjA7)	**I**SY**L**DAILGAVVK**V**
At2g21530	Protein of unknown function, contains FHA domain	**I**GR**L**PEKADVVIP**V**
At2g35490	Putative plastid-lipid associated protein (PAP)	**I**PL**L**AAGSTPLLK**V**
At2g36145	Unknown protein	**I**DC**L**VFQTTENGV**V**
At2g43560	FKBP-type peptidyl-prolyl isomerase (AtFKBP16-3)	**I**SN**L**SSRREAMLL**V**
At3g04790	Putative ribose-5-phosphate isomerase	**I**GK**L**LSSGELYDI**V**
At3g46780	Protein of unknown function (AtPTAC16)	**I**AS**L**VADIFANTA**V**
At4g39710	Putative component of lumen subcomplex of chloroplast NDH (AtFKBP16-2)	**I**RG**L**DQGILGGEG**V**
At5g39830	DegP-type serine protease (AtDeg8)	**I**SG**L**NRDIFSQTG**V**
At5g52970	Thylakoid lumen 15.0 kDa protein of unknown function	**I**AN**L**EKDTGFKLR**V**
At5g67030	Zeaxanthin epoxidase precursor (AtLOS6/AtABA1)	**I**NG**L**VDGISGTWY**V**

Overall, of the 32 putative cargo proteins identified, 14 are involved in photosynthesis, including five members of the light harvesting complex (LHC: AtLHCb6/At1g15820, AtOHP2/At1g34000, AtLHCb4.2/At3g08940, putative Chl a/b binding protein/At4g17600, AtLHCb4.1/At5g01530), four components of photosystem II (PSII) (AtLPA1/At1g02910, AtPsbS/At1g44575, AtPsbW/At2g30570, putative PsbT subunit/At1g51400), four components of photosystem I (PSI) (AtNDF6/At1g18730, AtPyg7/At1g22700, AtNDF2/At1g64770, AtFKBP16-2/At4g39710) and a putative subunit of the cytochrome *b_6_f* complex (putative subunit IV Cyt *b_6_f*/At2g26500). Another four are involved in transport, as components of Sec and SRP pathway translocases (AtSCY1/At2g18710, AtAlb3/At2g28800, AtSECe1/At4g14870) and a potassium channel protein (AtTPK3/At4g18160).

**Figure 3:**
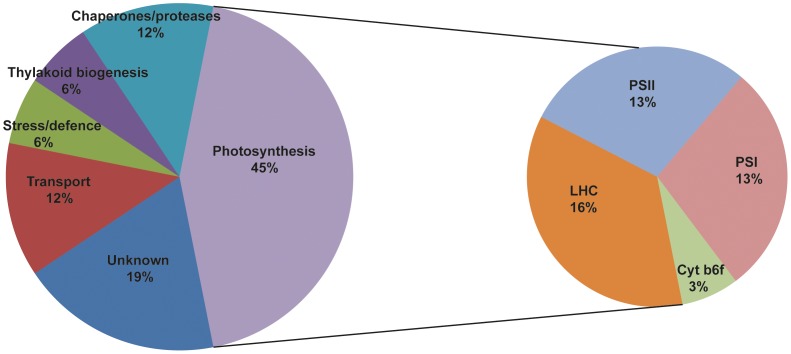
Distribution of functions of putative cargo proteins in Arabidopsis chloroplasts. As shown by the pie chart to the left, nearly half (45%) of the 32 putative cargo proteins are involved in photosynthesis, 19% have unknown functions, 12% are involved in transport, 12% are chaperones/proteases, 6% are involved in thylakoid biogenesis and 6% in stress responses/defense. The pie chart to the right shows that of the 45% of cargo proteins that are photosynthesis-related 16% are LHC proteins, 13% are PSII-related, 13% PSI-related and 3% components of the cytochrome b_6_f complex.

The remaining predicted cargo proteins are involved in thylakoid biogenesis (AtFCL/At1g03160, AtIM/At4g22260), stress and defense (putative ribose-5-phosphate isomerase/At3g04790, AtLOS6/At5g67030), proteases or chaperones (AtDjA7/At1g80030, AtFKBP16-3/At2g43560, AtDeg8/At5g39830, AtFtsH8/At1g06430), or have either unknown or unconfirmed functions (At4g31560, At2g21530, At2g36145, At3g46780, At2g35490, At5g52970) (Figure 3).

### Tethering Factors

When a loaded vesicle approaches the thylakoid membrane (acceptor membrane) tethering factors anchor it and prepare it for fusion by rearranging the SNARE complex(es). Possibly relevant motifs and domains of tethering factors found in yeast and human cytosol were scanned using Prosite, but no proteins containing the same motifs or domains in the chloroplast dataset were retrieved (Figure 1A). However, as tethering factors are a diverse group of proteins, we searched Prosite and Pfam for domains that could function as parts of tethering factors. Two domain profiles were retrieved: the GRIP domain (PS50913, PF10375), and RINTI/TIP20 domain profile (PS51386, PF04437). When the TAIR dataset was scanned for these two profiles no proteins with a GRIP domain and chloroplast location were identified, but one protein (AtMAG2/At3g47700) with a TIP20 domain was predicted to be chloroplast (Figure 1A) localized using WoLF PSort and PredSL (Table 5). Proteins containing a TIP20 domain form a multi-subunit tethering complex with two other proteins (Dsl1 and Sec20). We identified no homologues of these two other subunits in the chloroplast, although putative homologues have been previously identified in Arabidopsis [86].

**Table 5:** Putative chloroplast-localized tethering components.

Accession No.	Name (ARAMEMNON)	SigConsens (ARAMEMNON)			Comment	Localization (TAIR)	Chloro-plast 2010
		Chl.	Mt.	Sec. path.			
At5g16300	Putative COG1-like component	8.3	0	0	Chl., (MultiP, PCLR, PredSL, PProwler, SLPLocal, WoLF PSORT)	Cytosol, Golgi	-
At4g24840	Putative COG2-like component	8.5	2.2	0	Chl., (ChloroP, PCLR, PProwler, PredSL, SLPLocal, WoLF PSORT)	Golgi, Vacuole	Chl.
At1g73430	Putative COG3-like component	13	1.6	0	Chl., (ARAMEMNON)	Chl., Cytosol, Golgi	Chl.
At4g01400	Putative COG4-like component	5.4	11.3	0	Chl., BaCelLo, ChloroP, PCLR, PredSL, SLPFA, WoLF PSORT)	Cytosol, Golgi, Mt.	–
At1g67930	Putative COG5-like component	20.9	0	2.2	Chl., (ARAMEMNON)	Chl., Cytosol	Chl.
At1g31780	Putative COG6-like component	5.9	1.6	0	Chl., (MultiP, Predator, PredSL)	Cytosol, Golgi	–
At1g07725	AtExo70H6	5.4	0	0.4	Chl., (AdaBoost, BaCelLo, Plant-mPloc, PredSL, WoLF PSORT)	Exocyst	–
At2g28650	AtExo70H8	11.6	0	4.2	Chl., (ARAMEMNON)	Exocyst	Chl.
At2g39380	AtExo70H2	11.1	1	1.1	Chl., (ARAMEMNON)	Exocyst	Chl.
At3g55150	AtExo70H1	13.4	2.4	2.6	Chl., (ARAMEMNON)	Cytosol, Exocyst, Nucleus	Chl.
At5g59730	AtExo70H7	10.4	0	0	Chl., (ARAMEMNON)	Cytosol, Exocyst, Nucleus	Chl.
At3g47700	Protein involved in transport of seed storageproteins between ER and Golgi (AtMAG2)	8.3	0	3.6	Chl., PredSL, WoLF PSORT)	Chl., ER	–
At3g18480	AtCASP	5.3	0	0	Chl., (PredSL, TargetP)	Golgi, Endosome	Chl.

SigConsens, consensus prediction of subcellular localization; Chl., chloroplast; Mt., mitochondria; Sec. path., secretory pathway; Chl. loc., chloroplast localization; MS, mass spectrometry; ER, endoplasmatic reticulum; Chloroplast 2010, predicted chloroplast localized protein (http://www.plastid.msu.edu/index.html).

Several tethering protein homologues have been classified in Arabidopsis [86], [87]. They can be assembled into multi-subunit complexes, including the Conserved Oligomeric Golgi (COG) complex and the exocyst complex. By searching all the tethering factors in ARAMEMNON (Figure 1C), it was predicted that six of the total of eight COG complex subunits were chloroplast localized: COG1-like/At5g16300, COG2-like/At4g24840, COG3-like/At1g73430, COG4-like/At4g01400, COG5-like/At1g67930, COG6-like/At1g31780. The strongest of these ARAMEMNON predictions were for COG3-like and COG5-like subunits (Table 5). The other, exocyst, multi-subunit complex is composed of Sec3, Sec5, Sec6, Sec8, Sec10, Sec15, Exo70 and Exo84. Interestingly there are 23 homologues of Exo70 in Arabidopsis according to [88]. We found five of these Exo70 homologues (AtExo70H1/At3g55150, AtExo70H2/At2g39380, AtExo70H6/At1g07725, AtExo70H7/At5g59730 and AtExo70H8/At2g28650), except AtExo70H6m, to be strongly predicted to be chloroplast-localized by ARAMEMNON (Table 5). In addition, we found that one of the coil-coiled tethering factors, AtCASP/At3g18480 [89], could have a potential transit peptide (PredSL, TargetP) (Table 5). Multiple alignments with the closest homologues in human and yeast showed that AtCASP has an extra stretch of amino acids in the N-terminal side, suggesting targeting to the chloroplast (Figure S4).

t-SNAREs and v-SNAREsSNAREs are important for vesicle fusion and are often divided into a group that reside on the vesicle (v-SNARE) and another that reside on the target membrane (t-SNARE). Starting with the yeast dataset and using Prosite we identified a t-SNARE domain (PS50192), and a scan of the Arabidopsis chloroplast proteins identified two with this domain, the first being a synaptosomal-associated protein (SNAP), AtSNAP33/At5g61210 (Table 6). Pfam predicts AtSNAP33 to have two SNARE-related domains, SNAP-25 (PF00835) and SNARE (PF05739), supporting its role as a t-SNARE. Its chloroplast localization is predicted by TargetP, PCLR and PredSL and further supported by a previous proteomic analysis using SUBA [83]. Interestingly, two other SNAP proteins, AtSNAP29 and AtSNAP30, are also reportedly present in Arabidopsis [90] and when aligned to the putatively chloroplast-localized AtSNAP33 they show relatively weak conservation in the N-terminal side, indicating the presence of a transit peptide of AtSNAP33 (Figure S5).

**Table 6:** Putative chloroplast-localized SNARES and SNARE-associated proteins.

Accession No.	Name (ARAMEMNON)	SigConsens (ARAMEMNON)			Comment	Localization (TAIR)	Chloro-plast 2010	Chl. loc. (MS/MS)
		Chl.	Mt.	Sec. Path.				
At5g16830	Vesicle transport syntaxin-type t-SNARE protein (AtSYP21/AtPEP12)	7.6	1.8	3.8	Chl., (ChloroP, MultiP,PCLR, PProwler, TargetP)	Late endosome, Trans Golgi network	Chl.	
At5g61210	SNAP-25-type t-SNARE protein (AtSNAP33)	3.7	0.5	0	Chl., (PCLR, PredSL,Target P)	Chl., PM, Nucleus	Chl.	[83]
At1g04760	R-SNARE domain protein, synaptobrevin (AtVAMP726)	0	0	7.5	Chl., (BacelLo, WoLFPSORT)	Chl., Endosome, PM	Chl.	[83]
At4g05060	Putative protein trafficking vesicle-associated protein	8.3	0.1	0	Chl., (ChloroP, PCLR, PProwler, PredSL, TargetP)	Chl., PM	Chl.	
At1g22850	Putative vesicle transport SNARE-associated protein	14.2	8.5	4.0	Chl., (ARAMEM-NON)	Chl.	Chl.	[82]

SigConsens, consensus prediction of subcellular localization; Chl., chloroplast, Mt., mitochondrion; Sec. path., secretory pathway; Chl. loc., chloroplast localization; MS, mass spectrometry; ER, endoplasmatic reticulum; PM, plasma membrane; Chloroplast 2010, predicted chloroplast localized protein (http://www.plastid.msu.edu/index.html).

The second protein identified as a putative t-SNARE, AtSYP21/At5g16830, has two domains – the t-SNARE domain profile (PS50192 and PF05739) and the syntaxin domain profile (PS00914 and PF00804) – and has been named AtSYP21 but also referred to as AtPEP12 [90]. It has a transit peptide and is predicted to be localized in the chloroplast by ChloroP, MultiP, PCLR, PProwler and TargetP, but experimental support for this is lacking (Table 6). When aligning AtSYP21 (At5g16830) with its closest homologues in yeast and humans it shows no conservation at the N-terminal, where it has an extra stretch of amino acids (Figure S6), giving further support for a chloroplast localization via a transit peptide.

A Prosite scan of the TAIR chloroplast dataset (Figure 1) to find v-SNAREs identified AtVAMP726/At1g04760 (Table 6) with three domain profiles (v-SNARE, PS50892: longin, PS50859; synaptobrevin, PS00417), whereas Pfam only identified the longin (PF13774) and synaptobrevin (PF00957) domain profiles. This protein has no obvious transit peptide, but has been shown by BacelLo, WoLFPSORT and proteomic analysis to localize in the chloroplast [83].

### SNARE-associated Proteins

SNARE-associated proteins are believed to assist SNAREs at fusion [91], [92]. Two proteins with SNARE-associated features were found to be chloroplast localized. One of these (At1g22850) has a known domain profile of SNARE-associated Golgi proteins, according to Pfam (PF09335). It has a strongly predicted chloroplast location, according to the ARAMEMNON consensus score, and an extra stretch of amino acids when aligned with Arabidopsis SNARE-associated homologues (Figure S7). Furthermore, previous proteomic analysis supports a chloroplast localization for this protein [82].

The second predicted chloroplast-localized SNARE-associated protein is the Putative vesicle associated protein (VAP)/At4g05060, which has a transit peptide and chloroplast location predicted by PredSL, ChloroP, PProwler, PCLR and Target P. When aligned with yeast, human and one of the closest Arabidopsis cytosol homologues, the Putative VAP/At4g05060 shows an N-terminal stretch of the protein that could indicate a transit peptide (Figure S8). The Putative VAP/At4g05060 has a major sperm protein (MSP) domain profile (PS50202) according to Prosite (Figure 1, Table 6). An MSP domain has also been found in a mammalian protein called VAMP associated protein 33 (VAP33), where it causes binding to a v-SNARE (synaptobrevin/VAMP) and is strongly associated with vesicle fusion [92]. These findings suggest that the putative vesicle-associated protein could play the same role in chloroplasts.

### Rab GTPases

Rab GTPases (Rabs) usually facilitate vesicle tethering and fusion, but also reportedly assist in the vesicle budding process [28]. After running the yeast dataset of vesicle proteins in Prosite we found one Rab domain (PS51419) that, according to the TAIR chloroplast database and chloroplast prediction tools (Figure 1), is present in three proteins that could function as Rab GTPases in chloroplasts: AtRabA5e/At1g05810, AtRabB1c/At4g35860 and AtRabF1/At3g54840 (Table 7). A chloroplast location for AtRabA5e and AtRabF1, but not AtRabB1c, is further supported by ARAMEMNON.

**Table 7:** Putative chloroplast localized Rab GTPase proteins.

Accession No.	Name (ARAMEMNON)	SigConsens (ARAMEMNON)			Comment	Localization (TAIR)	Chloro-plast 2010	Chl. loc. (MS/MS)
		Chl.	Mt.	Sec. path.				
At1g05810	Putative RAB-A-class small GTPase (AtRAB-A5e)	17	0.9	5.5	Chl., (ARAMEM-NON)	Chl.	Chl.	
At4g35860	Putative RAB-B-class small GTPase (AtRAB-B1c)	0	0	5.5	–	Chl., Golgi	–	[82]
At3g54840	Putative RAB-F-class small GTPase (AtRAB-F1/AtARA6)	12.6	0	3.9	Chl., (ARAMEM-NON)	Chl., Endosome, Golgi	Chl.	

SigConsens, consensus prediction of subcellular localization; Chl., chloroplast; Mt., mitochondrion; Sec. path.; secretory pathway; Chl. loc., chloroplast localization; MS, mass spectrometry; Chloroplast 2010, predicted chloroplast localized protein (http://www.plastid.msu.edu/index.html).

Multiple alignments of the three putative Rab GTPases with their closest yeast and human homologues showed that AtRabA5e and AtRabF1 (but not AtRabB1c) also carry an extra N-terminal stretch of amino acids, possibly representing a transit peptide (Figures S9, S10, S11), supporting the ARAMEMNON findings.

### Rab GDFs and GAPs

To function properly Rab GTPases need both a GDP dissociation inhibitor (GDI) displacement factor (GDF), to catalyze dissociation of the GDI when bound to inactive GDP-bound Rab, and a GAP that promotes Rab activity. In Arabidopsis, PRA1 proteins function as GDFs and in total 19 PRA1 proteins are known in Arabidopsis [93]. When running the sequences of all the PRA1 proteins through the chloroplast localization predicting tools we identified nine of them as putatively localized in the chloroplast (Table 8). However, the ARAMEMNON predictions were especially strong (consensus scores ≥10) for five of them: AtPRA1.B4/At2g38360, AtPRA1.B2/At2g40380, AtPRA1.B3/At5g05380, AtPRA1.B1/At3g56110 and AtPRA1.B5/At5g01640 (Table 8).

**Table 8:** Putative chloroplast localized Rab GTPase-activating proteins and Rab receptor proteins.

Accession No.	Name (ARAMEMNON)	SigConsens (ARAMEMNON)			Comment	Localization (TAIR)	Chloro-plast 2010
		Chl.	Mt.	Sec. path.			
**Putative Rab GTPase-activating proteins (GAPs)**							
At5g53570	Unknown Function	13.1	3.1	3.7	Chl. (ARAMEMNON)	Intracellular, Golgi	Chl.
At4g13730	Rab GAP Putative	12.5	0	0	Chl., (ARAMEMNON)	Chl., Golgi, Intracellular,	Chl.
At3g49350	Unknown Function	13.4	5	2.1	Chl., (ARAMEMNON)	Chl.	Chl.
At5g24390	Unknown Function	9.4	9.5	3.4	Chl., (BaCelLo, ChloroP, MultiP, PCLR, plant-mPloc, PProlwler, SLPLocal, TargetP, WoLF PSORT)	Chl., Intracellular	Chl.
At5g57210	RAB GAP Putative	3.7	0	0	Chl., (Target P)	Chl., Intracellular, Nucleus	Chl.
At2g19240	RAB GAP Putative	4.2	0	0.6	Chl., (SLPLocal, TargetP)	Chl., Intracellular, PM	Chl.
**Putative Rab receptors**							
At2g38360	Putative Rab-type GTPase receptor (AtPRA1.B4)	20.6	0	0.4	Chl., (ARAMEMNON)	Chl., Cytosol, ER, Golgi	Chl.
At2g40380	Putative Rab-type GTPase receptor (AtPRA1.B2)	17.4	0	3.2	Chl., (ARAMEMNON)	Chl., Cytosol, ER, Golgi	Chl.
At3g13710	Putative Rab-type GTPase receptor (AtPRA1.F4)	5.9	0	2.5	Chl., (TargetP)	Chl., ER	Chl.
At5g05380	Putative Rab-type GTPase receptor (AtPRA1.B3)	12.1	1.1	0	Chl., (ARAMEMNON)	ER	Chl.
At3g13720	Putative Rab-type GTPase receptor (AtPRA1.F3)	8.1	0	3.4	Chl., (IPSORT, PCLR, PredSL, SLPLocal, YLoc)	Chl., ER	–
At3g56110	Putative Rab-type GTPase receptor (AtPRA1.B1)	15.4	0	0.3	Chl., (ARAMEMNON)	ER, Golgi	Chl.
At5g01640	Putative Rab-type GTPase receptor (AtPRA1.B5)	11.8	0	2.9	Chl., (ARAMEMNON)	ER	Chl.
At5g07110	Putative Rab-type GTPase receptor (AtPRA1.B6)	9.2	1.2	5.6	Chl., (BaCelLo, ChloroP, IPSORT, PredSL, SLPLocal, TargetP, YLoc, MultiP)	ER, Golgi	Chl.
At5g56230	Putative Rab-type GTPase receptor (AtPRA1.G2)	9	0	4.1	Chl., (BaCelLo, ChloroP, IPSORT, PCLR, SLPLocal, SLPFA, TargetP)	Chl., ER	Ch.l

SigConsens, consensus prediction of subcellular localization; Chl., chloroplast; Mt., mitochondria; Sec. path., secretory pathway; Chl. loc., chloroplast localization; ER, endoplasmatic reticulum; Chloroplast 2010, predicted chloroplast localized protein (http://www.plastid.msu.edu/index.html).

Yeast contains a Rab GAP, called GAP for Ypt (GYP) that contains a Tre-2/Bub2/Cdc16 (TBC) domain (PS50086, PF00566), which is important for catalytic activity of Rab GAPs [38]. When scanning the chloroplast dataset in Prosite we found six putative Rab GAP proteins containing the TBC domain (Table 8). All six of these putative Rab GAP proteins were predicted to be located in the chloroplast by ARAMEMNON, three strongly (with consensus scores ≥10), including two unknown proteins, At5g53570 and At5g53570, and one putative Rab GAP/At4g13730 (Table 8).

### Reticulons

Reticulons have suggested involvement in the late stage of vesicle transport and have been shown to interact with proteins regulating vesicle fusion and Rab-regulated intracellular trafficking [42]. We found three proteins that could act as reticulons in the Arabidopsis chloroplast: reticulon type At5g58000, At4g28430 and At2g20590 (Table 9). Both Prosite and Pfam identified reticulon domains (PS50845, PF02453) in these proteins when scanning the chloroplast protein dataset retrieved from TAIR (Figure 1). Furthermore, ARAMEMNON predicted them to be chloroplast localized. Multiple alignments with their closest homologues in Arabidopsis and yeast also showed that an extra N-terminal sequence in the putative chloroplast reticulons might be a transit peptide responsible for chloroplast targeting (Figure S12), strongly indicating that At5g58000, At4g28430 and At2g20590 are located in chloroplasts.

**Table 9:** Putative chloroplast localized reticulon proteins.

Accession No.	Name (ARAMEMNON)	SigConsens (ARAMEMNON)			Comment	Localization (TAIR)	Chloro-plast 2010
		Chl.	Mt.	Sec. path.			
At5g58000	Putative Reticulon-type ER-associatedprotein of unknown function	10.8	0	0	Chl., (ARAMEMNON)	Chl., ER	Chl.
At4g28430	Putative Reticulon-type ER-associatedprotein of unknown function	14,1	3.4	0	Chl., (ARAMEMNON)	Chl., ER	Chl.
At2g20590	Putative Reticulon-type ER-associatedprotein of unknown function	15.2	0	0	Chl., (ARAMEMNON)	Chl., ER	Chl.

SigConsens, consensus prediction of subcellular localization; Chl., chloroplast; Mt., mitochondria, Sec. path., secretory pathway; Chl. loc., chloroplast localization; ER, endoplasmatic reticulum; Chloroplast 2010, predicted chloroplast localized protein (http://www.plastid.msu.edu/index.html).

## Discussion

### Lipid Composition in Chloroplasts Versus ER

The donor membrane that is the starting point of the cytosolic COPII vesicle transport system is the ER. The corresponding donor membrane in the chloroplast, the inner envelop membrane, has a high proportion of glycolipids (ca. 85% of the total amount of polar lipids; [94]. In contrast, the ER membrane lacks glycolipids, but has a very high proportion of phospholipids, such as phosphatidylinositol (PI) and phosphatidylcholine (PC). The head group of PIs is wedge-shaped and important for membrane curvature, whereas the head group of PCs is cylindrical [95]. The chloroplast inner envelope only contains around 1% of PI [94] whereas the ER membrane contains about 20% PI [96]. Thus, the initiation of vesicle assembly and budding involves different lipids in the inner envelope membrane than in cytosolic vesicle transport. However, the glycolipid monogalactosyl diacylglycerol (MGDG) in the chloroplast envelope has a wedged-shaped form, similarly to PI, which has been reported to transported in vesicles from the inner envelope membrane to the thylakoid acceptor membrane [8], [10]. It has been suggested that if MGDG synthesis occurs on the non-stromal side in the inter membrane space then the wedge-shaped MGDG will be on the stromal side, facilitating vesicle budding [10]. Thus, if the wedge shape is important for vesicle formation then MGDG could fulfill this role that PI possesses in the ER.

SEC14 proteins are cytosolic proteins reported to participate in post-Golgi transport, playing an important role in vesicle biogenesis, maintaining a high PI to PC ratio in the membrane where they are located [97]. Notably, four SEC14-like proteins were predicted to be chloroplast localized (Table 1). [96]. The Arabidopsis dynamin-like 2 protein (ADL2a) has been shown to bind specifically to a phosphorylated form of PI (phosphatidylinositol 4-phosphate) in chloroplasts and has suggested involvement in vesicle budding at the chloroplast envelope [98]. Regardless of the different lipid compositions in the ER and chloroplast inner envelope MGDG could have a similar role in the chloroplast as PI in the ER, promoting membrane curvature with its wedged shape where the role of SEC14 could be to concentrate the PI in the chloroplast, facilitating vesicle formation.

### Vesicle Initiation - CPSAR1 and its GEF

Although CPSAR1 has been characterized and shown to be involved in thylakoid biogenesis [9], its origin and function have been debated [80], [81], [99] since it has similarities to bacterial Obg proteins, which have various functions. For instance, CPSAR1 (AtObgC) has been implemented to also play a vital role for chloroplast ribosome biogenesis [80], [99] in addition to its role in vesicle transport [9]. CPSAR1 has a GTPase domain with GTP hydrolysis activity [9], [80], [81], and the presence of CPSAR1 in a soluble and membrane bound form [9] indicates similarities with small GTPases including cytosolic Sar1 of yeast and Arabidopsis. However, CPSAR1 contains a unique extended N-terminus of approximately 200 amino acids compared to yeast and Arabidopsis Sar1, and Obg proteins [19]. In Sar1 the N-terminus interacts with the membrane, which is not yet resolved for CPSAR1. If the extended N-terminus reflects the different lipid composition exposed to CPSAR1 for membrane interaction or the suggested function for ribosomal biogenesis is currently not known.

Regardless of its origin and differences from cytosolic Sar1 CPSAR1 could have similar functions, e.g. involvement in vesicle transport and interaction with a GEF. In our study we found one chloroplast-localized GEF similar to Sec12, AtPHF1 (Table 1). AtPHF1 is a phosphate transporter traffic facilitator and considered a Sec12-like protein located on the ER [100], [101]. It has not been reported to act as a GEF for Sar1, but the possibility that it may interact in such a manner with CPSAR1 in chloroplasts remains to be elucidated.

**Figure 4:**
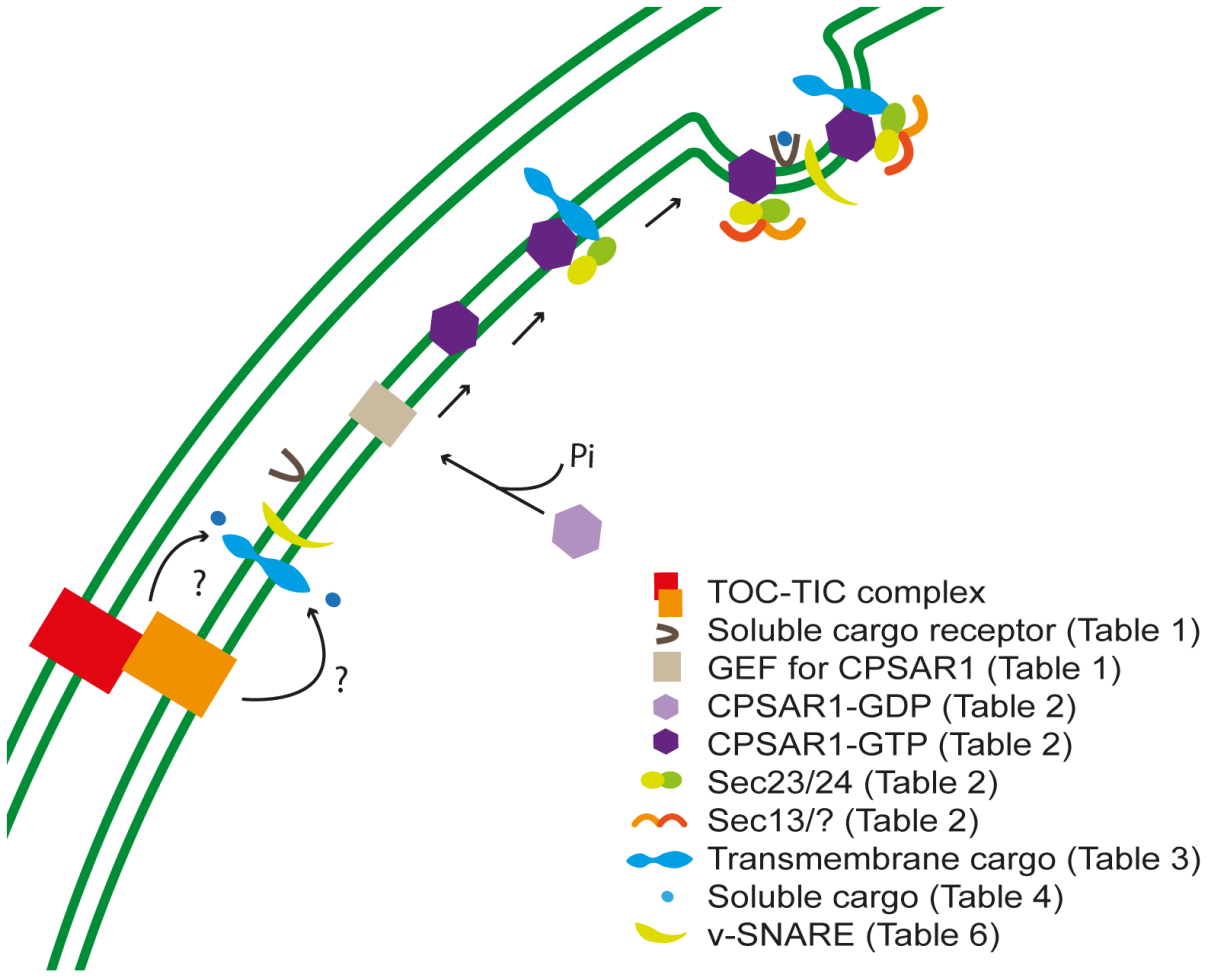
Model for vesicle initiation and budding in Arabidopsis chloroplasts. Nucleus-encoded transmembrane or soluble cargo proteins enter the chloroplast via the TOC/TIC machinery and by an unknown process approach cargo protein receptors (soluble cargo proteins) or are integrated into the inner envelope membrane (transmembrane cargo proteins). Vesicle initiation involves activation of CPSAR1 in its inactive state (CPSAR1-GDP) by a GEF protein similar to Sec12, causing it to attach to the inner envelope membrane in its active state (CPSAR1-GTP). The budding process involves recruitment of two coat proteins, Sec23/24 and Sec13, prior to scission.

Based on our findings we propose that AtPHF1 may be targeted to both the ER and the chloroplast. The presence of membrane contact sites between ER and chloroplasts, in the form of plastid associated membranes (PLAM) [102] could be one possible scenario facilitating AtPHF1 being located in these different localities. Thus, AtPHF1 could function as a phosphate transporter traffic facilitator in the ER but as a CPSAR1-activating GEF in chloroplasts (Figure 4). In accordance with this hypothesis, it is known that some proteins are present in several compartments of cells due to dual targeting [103]. The majority of proteins being dual targeted are observed between chloroplast and mitochondria because of parallel evolutionary history [104], but dual targeting is not restricted to these organelles. For instance: the filament-forming protein FtsZ is present in both chloroplasts and cytoplasm of moss *Physcomitrella patens* [105]; an aminoacyl-tRNA synthetase is present in the cytosol, mitochondria and chloroplasts of Arabidopsis [106]; the plant glutamate receptor AtGLR3.4 is localized in both the plasma membrane and plastids of Arabidopsis and tobacco [107]; the cytochrome *b5* protein is present in both ER and mitochondria in cauliflower (*Brassica olracea*) [108]; RB60 is an atypical protein disulfide isomerase (PDI) that ends up in both ER and chloroplasts in *Chlamydomonas reinhardtii* [109]; the ADL1a protein has been is found in both thylakoids [110] and the cytosolic secretory system of Arabidopsis [111]; the ADL2a protein is localized in chloroplast envelopes [112], peroxisome and mitochondria in Arabidopsis [113], [114]; a potassium channel protein TPK3 is found in the vacuole [115] and thylakoids in Arabidopsis [116]. Thus, the examples above clearly show that the prediction in our study of proteins being dual targeted can be valid although future experimental tests are necessary to validate the data.

### Vesicle Coat Assembly and Budding

**Figure 5:**
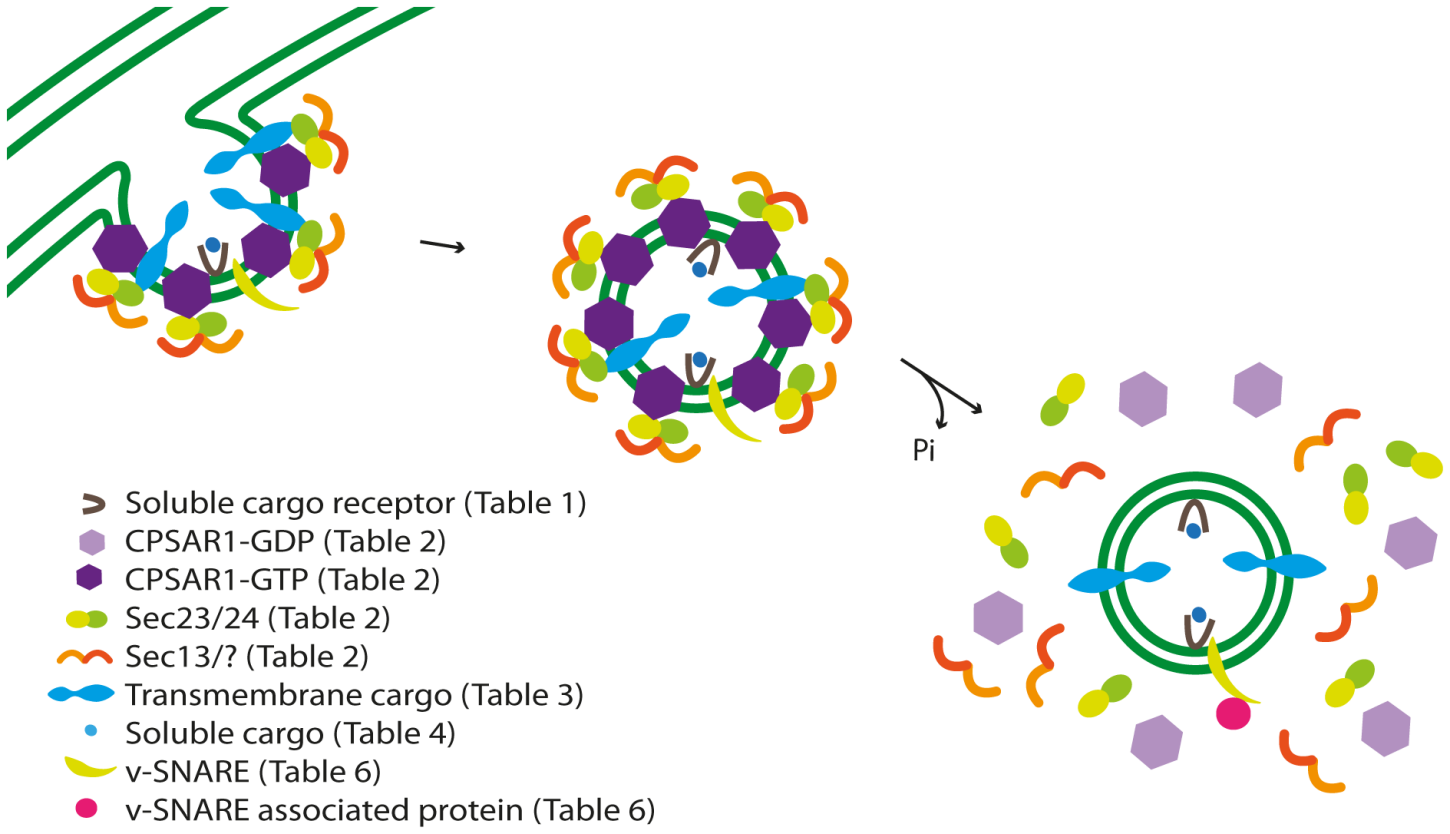
Model for vesicle scission and uncoating in Arabidopsis chloroplasts. When the two coats are in place, together with cargo receptors and possibly cargo proteins, the coat buds from the inner envelope membrane. Soon after budding the vesicle loses its coat, as CPSAR1-GTP dissociates and becomes inactive, in the CPSAR1-GDP state. The uncoated vesicle, also harboring v-SNARE and v-SNARE associated proteins for the forthcoming tethering process, moves towards the thylakoid membrane.

Activation of Sar1 leads to recruitment of the Sec23–Sec24 complex to nascent COPII vesicles, Sec23 and Sec24 acting as a GAP for Sar1 hydrolysis [117] and selection of cargo proteins [16], respectively. Homologues to these proteins were predicted in our study, thereby supporting the previous putative Sec23 and Sec24a/b findings (Table 2) [19]. The Sec24 amino acid sequence responsible for binding cargo proteins has been conserved between species [17]. Thus, Sec24 homologues could be responsible for selecting cargo proteins, indicating that vesicles could transport cargo proteins in addition to lipids, in chloroplasts (Figure 5).

The outer layer of the COPII vesicle coat is composed of a Sec13–Sec31 complex, which helps in connecting adjacent coat complexes [118]. We detected two Sec13 chloroplast homologues, but none for Sec31. Previous, conflicting results support the presence of only one Sec13 homologue and two Sec31 homologues (Sec31a/b) [19]. When aligning these proteins with their yeast and Arabidopsis homologues (no alignments were included in the previous study by Andersson and Sandelius [19]) the Sec13-like function of the Sec13 homologues was supported, but the Sec31a/b homologues showed no indication of a specific Sec31 function, although we also predicted them to be chloroplast localized. It could be argued that only Sec13 is needed to form the outer layer of vesicle coating in the chloroplast, or that a true Sec31 still remains to be identified, whereas both Sec23 and Sec24 are present in the inner layer, the latter opening the possibility of cargo proteins being transported in chloroplast vesicles (Figures 4 and 5).

Although there is bioinformatics support for coating of vesicles inside chloroplasts, none of these components have been verified as yet, despite being known since publication of the study by Andersson and Sandelius [19]. Thus, there is a need to clarify unambiguously whether chloroplasts possess a vesicle transport system that is identical to the cytosolic system. Moreover, the main indications that most components of the cytosolic system have a cytosolic location, except Sec13, have been acquired through proteomic analysis [119], raising the possibility that some may be dual-targeted since the chloroplast homologues clearly have a predicted transit peptide.

### Vesicle Cargo Receptor Proteins

Three putative receptors for soluble cargo proteins in the chloroplast were identified: AtPATL1 and two SEC14-like proteins (Table 1). Although all were stated to be in the chloroplast by TAIR only one was predicted to be in the chloroplast by TargetP; the SEC14-like protein At4g09160, indicating that the others lack a transit peptide. However, proteins can be targeted to the chloroplast envelope or thylakoid membrane without having a transit peptide [83]; known examples include AtGLR3.4, the translocon proteins of the outer envelope of the chloroplast membrane, AtToc33 and AtToc34, and ceQORH [107], [120], [121]. Thus, all three cargo receptors predicted here could be true chloroplast proteins, a possibility supported by previous proteomic analysis [83].

All three putative receptors contain two domains: a GOLD domain at the C-terminus and a CRAL-TRIO domain at the N-terminus. The GOLD domain is known from the P24 protein family, and is present in proteins from diverse species including plants, mammals and yeast. They are part of the vesicle transport system, involved in cargo protein selection and sorting in COPI and COPII vesicles [84], [122]–[124]. P24 of the cytosolic vesicle transport system contains four regions: an N-terminal GOLD domain in the ER lumen that interacts with the cargo protein; a coiled-coil domain that interacts with other P24 proteins to form tetramers; a transmembrane region and a C-terminal cytoplasmic region, mainly containing hydrophobic amino acids such as a dilysine motif or two or more basic amino acids, which interacts with COPI and COPII [37], [84]. Interestingly, the GOLD domain of the cytosolic vesicle transport system receptors is in the N-terminus, while it is in the C-terminus of the chloroplast cargo receptors.

The CRAL_TRIO domain is found in Sec14 proteins, where it is required for vesicle budding and biogenesis [97], [125]–[127]. During endocytosis a SEC14-like protein, containing both the CRAL_TRIO and GOLD domain, interacts with cargo proteins [128]. Thus, possibly the N-terminal domain interacts with lipids during vesicle budding and the GOLD domain select cargo proteins. It remains to be investigated how a receptor facilitates cargo transport without a transmembrane helix and if any interaction occurs with the coat proteins or not. Interestingly, this domain has also been found in GAP and GEF proteins in Ras- and Rho-GTPase family proteins [129]. That the cargo receptors have two domains, one known to select cargo proteins and the other to function in budding and as GAP or GEF, implies that AtPATL1 and the two SEC14-like proteins could have several functions in the chloroplast, all related to vesicular trafficking (Figures 4 and 5).

### Vesicle Cargo Protein Transport

**Figure 6:**
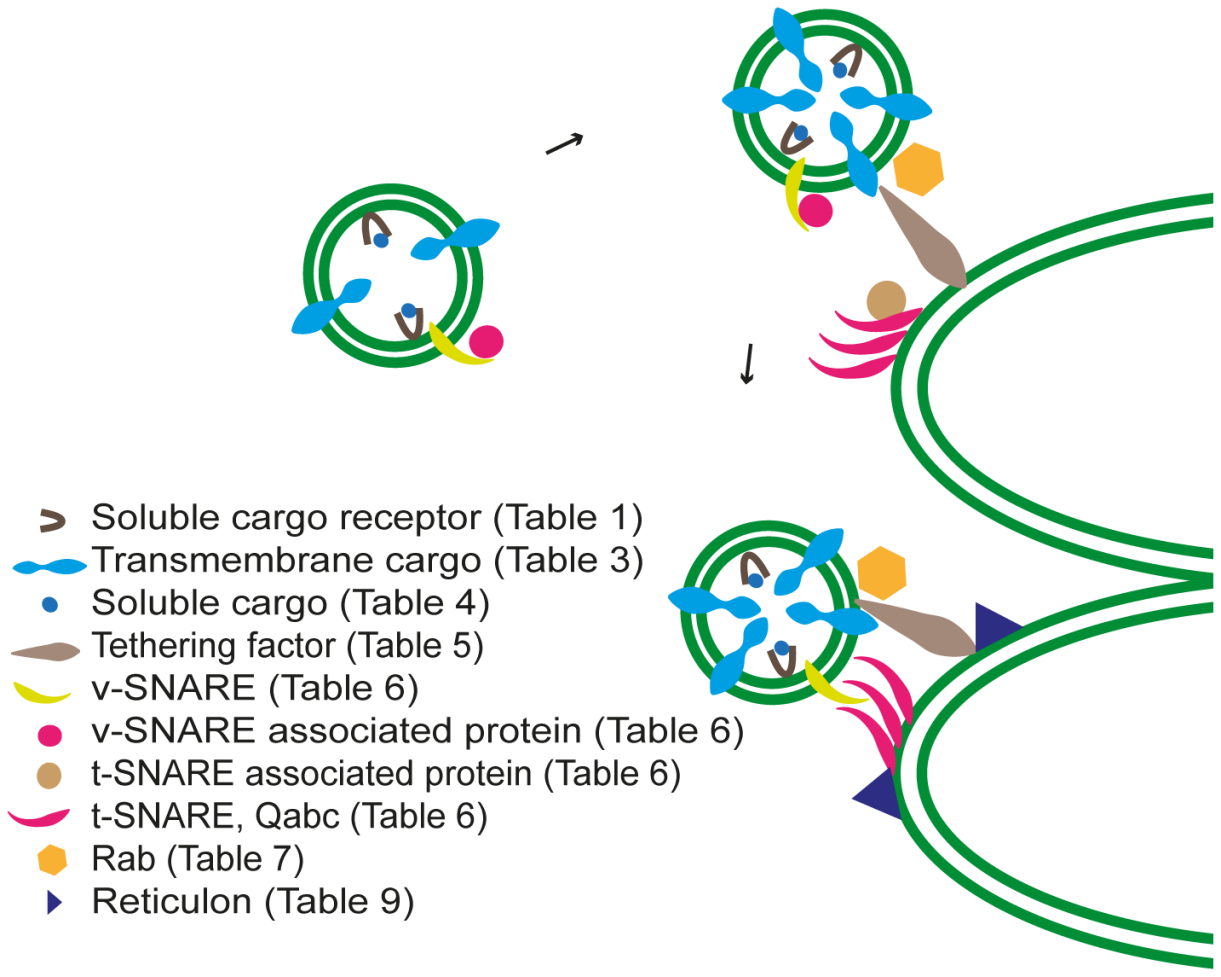
Model for tethering and docking in Arabidopsis chloroplasts. The uncoated vesicle moves towards the thylakoid membrane, and becomes tethered to the acceptor membrane by the combined actions of Rab and tethering factors. The v- and t-SNAREs assemble into a tight bundle with the assistance of v- and t-SNARE associated proteins.

**Figure 7:**
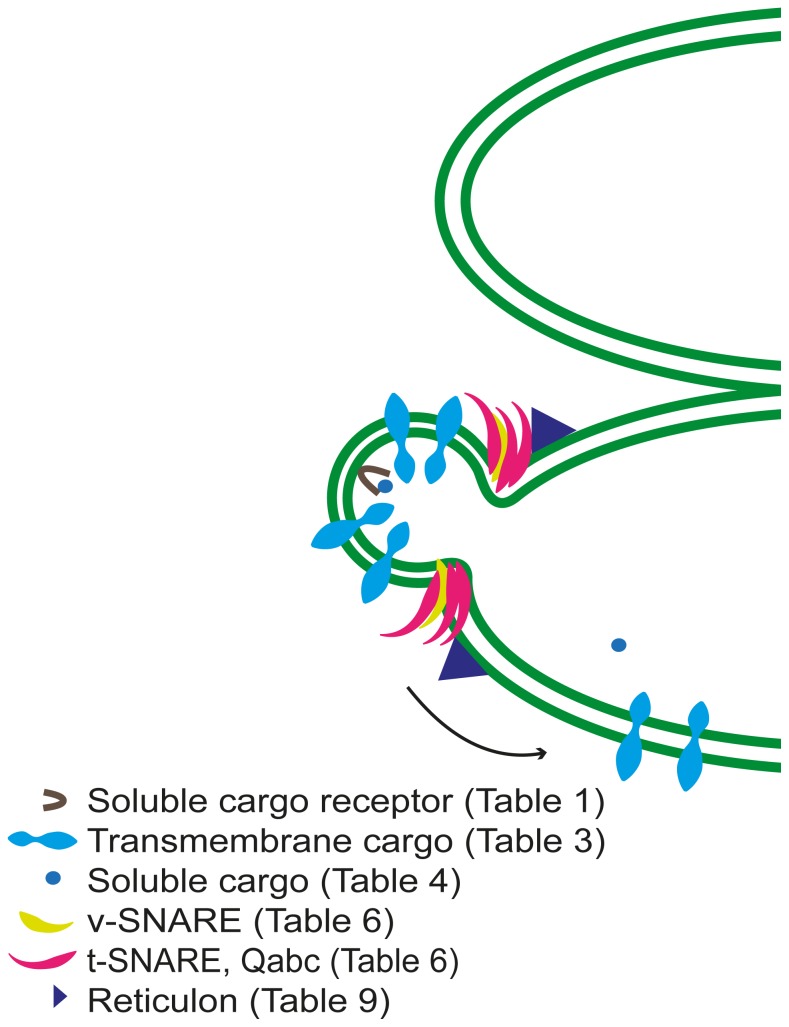
Model for vesicle fusion and cargo protein delivery to the thylakoid membrane in Arabidopsis. The lipids of the vesicle fuse with the thylakoid membrane lipids and the transmembrane cargo proteins are transferred to the thylakoid membrane, whereas the soluble cargo proteins are released from the cargo receptors and delivered to the lumen.

Transmembrane and soluble cargo proteins transported by the secretory pathway have an amino acid motif or signal required for inclusion in, for instance, COPII type vesicles [21], [22]. Most transmembrane cargos possess a diacidic signal in the cytoplasmic tail of their C-terminus [130]–[136]. In potassium channels of plants transported from the ER to the plasma membrane via the Golgi in a similar manner to COPII trafficking, a diacidic signal has been found that is important in various signal transduction pathways [17], [131]. Another common signal motif comprises a simple combination of two adjacent hydrophobic residues at or near the C-terminus [21], [137]. In plants a dihydrophobic residue signal is required for incorporation of AtP24 proteins in COPII vesicles, together with another signal, a dilysine motif at the C-terminal [124]. In *Cricetulus griseus* (hamsters), glycosyltransferases recognized by a dibasic motif in the N-terminus have been found to be transported as cargos in COPII vesicles [20]. In plants, in contrast, a single basic residue at the N-terminus may be sufficient for a protein to be carried as cargo in vesicles [138]. For soluble proteins to be transported using vesicles an ILV motif is critical as a cargo selector [21], [139]. Based on these signals we identified several possible cargo proteins involved in photosynthesis or thylakoid biogenesis, as transporters, proteases or chaperones, all located in the thylakoid membrane (Figure 3). We suggest that CPSAR1, with the help of other components, can select cargo proteins (Figures 4 and 5) that are then transported to the thylakoid membrane and used for thylakoid maintenance and photosynthesis (Figures 6 and 7).

Other proteins involved in thylakoid biogenesis, but also linked to chloroplast vesicle transport, are FZL [23], [140], THF1 [26], and VIPP1 [24], [141], [142]. FZL, one of the putative cargo proteins we identified, is a member of the dynamin superfamily, comprised of large GTPase proteins that participate in vesicle fission during endocytosis [140] and FZL has been suggested to be involved in chloroplast and thylakoid morphology and biogenesis being dual localized to the inner envelope and the thylakoids [23]. As we found several transmembrane cargo proteins with an diacidic motif that are involved in the PSII complex, and THF1 has suggested responsibility for PSII complex biogenesis [143], fusion of vesicles transporting PSII complex proteins might be facilitated by THF, which is important for thylakoid formation being localized both in stroma and thylakoids [23].VIPP1 is just like FZL associated with both the inner envelope and thylakoids, consistent with a trafficking function. In the absence of VIPP1 vesicle formation between inner envelope and thylakoid is abolished. However, the exact function of VIPP1 is unknown although a recent study concluded that VIPP1 could stimulate thylakoid reorganization to increase association of proteins and membrane regions housing the Tat translocon thus indirectly facilitate protein translocation using Tat [144]. However, if this increase of protein translocation involves vesicles is yet to be elucidated. The Tat and the Sec thylakoid targeting pathways facilitate luminal protein transport, and the Tat pathway can also transport folded proteins [5]. Four of the 12 identified luminal cargo proteins are predicted previously to be transported using either the Tat (AtFKBP16-3 and AtDeg8) or the Sec (AtFKBP16-2 and Thylakoid lumen protein) pathway (Figure 7; Table 3) [145], [146], whereas the other eight luminal cargo proteins use an undefined pathway meaning they could take a pathway other than the Sec or Tat pathway, possibly involving vesicle transport (Figures 6 and 7). However, if such vesicle transport is linked to Tat or interacting with VIPP1 is currently unknown.

Two other thylakoid protein targeting pathways have been previously defined, the SRP and spontaneous pathways that facilitate transport of thylakoid integral proteins [5]. Five LHC proteins were amongst the transmembrane cargo proteins (Figure 3; Table 3). On the basis of previous findings one can assume that light-harvesting chlorophyll binding (LHC) proteins could be transported via the SRP pathway [147]–[151] However, it was recently proposed that LHC proteins might be transported to the thylakoid in vesicles based on analyses using the Snowy Cotyledon 2 mutant (SCO2) [25]. Thus, the SCO2 chaperone interacts directly with the LHC protein but not with SRP pathway components and in the absence of SCO2 transport vesicles from the inner envelope to the thylakoids was perturbed [25]. Our results also suggest that both LHC proteins and chaperones could be cargo proteins and thus might be cooperatively transported in vesicles to the thylakoid.

Furthermore, most PSII transmembrane protein components (including two of our potential cargo proteins; AtPsbS and AtPsbW) can be transported spontaneously independently of the SRP pathway [152]. Altogether, the data suggest the presence of another pathway, possibly vesicle transport, in addition to the four already defined thylakoid protein targeting pathways. Furthermore, signals for cargo proteins in chloroplasts might differ from those in the cytosol e.g. there may be more specific signals than those defined for the cytosolic vesicle transport system, and if so they could not be found using our bioinformatics approach.

### Vesicle Fusion - Tethering Factors

Tethering factors form a bridge between vesicles and the target membrane and interact with SNAREs for correct fusion. Tethering factors are either oligomeric complexes (COG, Dsl1 and exocyst) or coiled-coil tethers, which can act as Rab effectors or Rab GEFs. In yeast and mammals oligomeric COG complexes are localized on the Golgi and Dsl1 on the ER, and they assist in COPI-mediated retrograde and anterograde transport, whereas exocysts play a role in secretion of vesicles at the plasma membrane [41]. COG, Dsl1 and exocyst complexes appear to have evolved from a single precursor and could therefore have similar functions [153], [154]. This is interesting since some, but not all, of the components of the COG, Dsl1 and exocyst complexes were found to be chloroplast localized. This suggests that a multi-subunit complex might not be required for tethering vesicles in the chloroplast, instead it could be accomplished by fewer tethering subunits, or due to homology in structure they could work synergistically in forming oligomeric complexes and tethering of the vesicles (Figures 6 and 7).

The only coiled-coil tethering factor found in chloroplasts was AtCASP, which has been previously characterized and shown to be Golgi-localized [87], [89]. AtCASP has been shown to be transported from ER in a COPII-dependent manner by possessing a diacidic motif on the cytoplasmic side [155], [156], and in humans CASP co-precipitates with Sec23, supporting a link to vesicle transport [157]. Although it is characterized as a Golgi resident protein its chloroplast prediction suggests that it could be dually targeted and may also help in tethering vesicles to the thylakoids (Figures 6 and 7). There are examples of proteins found in the thylakoids being also targeted to the cytosol, e.g. ADL1a is found in both thylakoids [110] and the cytosolic secretory system [111], and AtTPK3, despite having a low consensus score according to ARAMEMNON, has been found in the thylakoids [116] and the vacuole [158].

### Vesicle Fusion – SNAREs and SNARE-associated Proteins

Most of the chloroplast-localized proteins we found to be involved in fusion have previously been documented in the cytosolic vesicle transport system, suggesting that they are dually targeted, allowing them to function in chloroplasts as well as the cytosol. SNAREs are involved in docking of vesicles by assisting their fusion with the target membranes [159]–[161]. SNAREs form a superfamily of proteins, with 25 members in yeast [39], [162], 36 members in humans [39], [163] and more than 60 members in Arabidopsis [164], [165]. A shared characteristic of all SNAREs is the SNARE motif; an evolutionarily conserved stretch of 60–70 amino acids arranged in heptad repeats [166], [167]. Originally SNAREs were classified as v-SNAREs or t-SNAREs [161]. However, this terminology is not useful for describing homotypic fusion events, therefore they are now classified as Q-SNAREs, containing conserved glutamine residues or R-SNAREs, containing conserved arginine residues. Q-SNAREs are further classified as Qa, Qb and Qc SNAREs on the basis of amino acid composition [30], [166], [168]. Functional SNARE complexes that drive membrane fusion form parallel four-helix bundles, requiring one each of the Qa, Qb, Qc and R-SNAREs [39].

One of the chloroplast SNARE proteins identified, AtSNAP33 (Table 6), belongs to the SNAP25 protein family and contains two SNARE motifs, one each in the N- and C-terminals, joined by a flexible, palmitoylated linker. Members of this family of SNAREs act as both Qb and Qc t-SNAREs [39], [169]. AtSNAP33 refers to a subfamily of SNAP25s in Arabidopsis, and in a GFP fluorescence analysis AtSNAP33 was found to be dispersed in the cytosol [90]. SNAP25 family proteins are localized in the Golgi apparatus, plasma membrane and endosomes in mammalian cells, but only in the plasma membrane in yeast [169]. AtSYP21/AtPEP12 (Table 6) is another chloroplast-localized SNARE, which could function as a Qa t-SNARE, from a family of syntaxins. It is localized in the vacuolar membrane [90] and involved in post-Golgi trafficking in plants [170], [171]. Finally, a third chloroplast SNARE protein, AtVAMP726 (Table 6), is classified as an R v-SNARE, which is localized in the plasma membrane [90] and has a longin domain at the N-terminus. The longin domain helps in membrane fusion [172], [173]. AtVAMP726 also has a C-terminal domain, called synaptobrevin, which can be found in other SNARE proteins.

One of the SNARE-associated proteins we found, the Putative VAP (Table 6), has a major sperm protein (MSP) domain profile. This domain has been found in VAPs, e.g. VAP33, where it binds to the v-SNARE synaptobrevin/VAMP [92], [174]. Since the Putative VAP has the same domain it seems reasonable to assume that it also binds to SNAREs and functions as a SNAP. The second protein suggested to be a SNAP is the Putative SNARE Associated Protein (Table 6), the closest homologue of which in yeast being Tvp38, which co-localizes with the tig-2 t-SNARE [91], [175]. Tig-2 belongs to the syntaxin protein family and is involved in post-Golgi trafficking [176], [177], implying that the Putative SNAP also may also be localized with SNAREs and involved in vesicle transport.

Overall, from these data we suggest that AtSNAP33 acts as a Qbc t-SNARE, and AtSYP21/AtPEP12 as a Qa t-SNARE in association with the Putative SNARE-associated protein. These proteins could form three Qabc t-SNARE bundles on the thylakoid membrane (Figures 6 and 7). The v-SNARE AtVAMP726 could act as an R-SNARE on the vesicles, associating with the Putative VAP from the donor membrane containing a transport intermediate and allowing fusion with the target membrane by making the fourth bundle.

### Rabs and Reticulons

Rab GTPases appear to play diverse roles in vesicle transport, as they are reportedly involved in vesicle budding, motility, tethering and docking [28]. The Arabidopsis genome encodes 57 Rab proteins, divided into eight subfamilies (RabA to RabH) based on sequence similarities [32], [178]. We found three Rab proteins in the chloroplast, all classified as Rab GTPases in Arabidopsis [178]: AtRabA5e, AtRabF1, and AtRabB1c.

AtRabA5e is related to the mammalian Rab11 and Rab25, and the yeast YPT31/30, which operates between the endosome, the Golgi and the plasma membrane [32], suggesting it plays a role in transport events between the Golgi and plasma membrane [179]. However, AtRabA5E has not been observed in the cytosolic vesicle transport system, and its predicted transit peptide suggests a role in vesicle transport in chloroplasts. Interestingly, it was first predicted to be a chloroplast-localized Arf1 [19], but searches in Prosite showed it has a potential Rab domain profile, thus we consider it a Rab protein.

AtRabB1c is related to mammalian Rab2 [32], which is localized on *cis*-Golgi membranes and interacts with Golgi matrix proteins [180], [181]. Rab2 is also involved in the maturation of vesicular tubular clusters (VTCs), which are microtubule-associated intermediates in transport between the ER and Golgi apparatus [182]. AtRabB1C could be involved in retrograde or anterograde transport between the ER and Golgi, it has been localized in the ER and Golgi apparatus [183], and shown to have a role in early embryogenesis in plants [184].

AtRabF1 is most similar to Rab5 and Rab22 of mammalians and YPT51/YPT52/YPT53 of yeast, all of which are involved in endocytosis and endocytic-sorting pathways [28], [185], [186]. AtRabF has also been suggested to participate in the endocytosis pathway in plants [179]. AtRabF1 acts in association with SNAREs such as SYP121 and VAMP 127 [187], [188]. Thus, it can be assumed to work together with the putative SNAREs found in the chloroplast (Figure 6).

**Figure 8:**
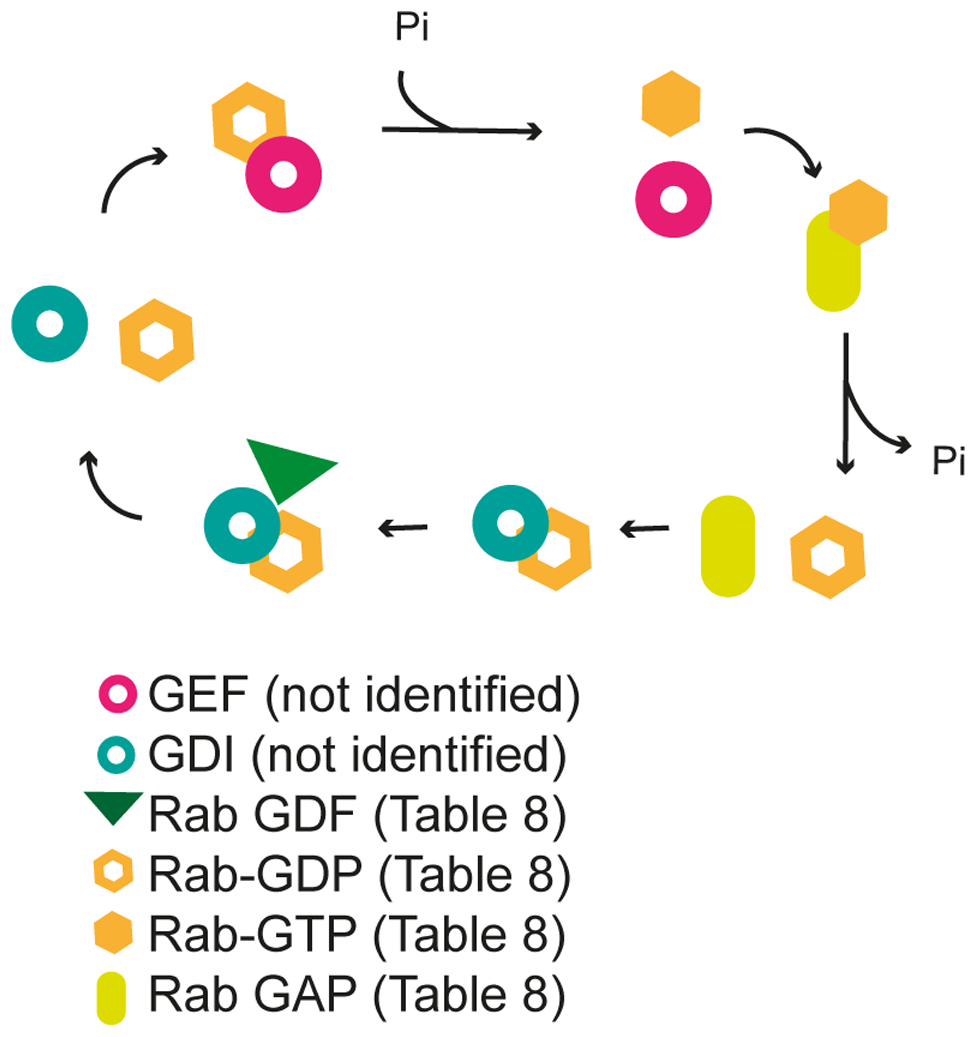
Rab cycling in Arabidopsis chloroplasts. Rab GTPase in its inactive, Rab-GDP, form is transformed into its active, Rab-GTP, form by an unidentified GEF in the stroma. A GAP promotes activity of the Rab GTPase to perform this action. To function properly the Rab GTPase needs a GDP dissociation inhibitor (GDI) displacement factor (GDF) that catalyzes dissociation of the GDI when bound to the inactive Rab-GDP.

As Rab GTPases cycle between active and non-active forms, proteins required to catalyze this cycle would be needed for them to function properly (Figure 8). We found several GAPs for hydrolysis of Rab and a GDF (Table 8), but failed to identify any GDI or GEF specific for Rab proteins. However, such proteins could be present, but without the characteristic features of their cytosolic counterparts (Figure 8).

The reticulon family of proteins is primarily associated with the ER and involved in vesicle trafficking in the cytosolic vesicle transport system. To date, 21 proteins have been found with a reticulon homology domain (RHD) in Arabidopsis, although very little is known about their subcellular localization and function [33], [189]. It has been suggested that a reticulon called RTN3 plays a role in the early secretory system between the ER and Golgi [35] and reticulon 1-C has been shown to form a complex with the SNAREs syntaxin 1, syntaxin 7, syntaxin 13 and VAMP2 [34]. In human cells, TBC120 (a Rab GAP for Rab1 and Rab2) interacts with a reticulon called RTN1 and both are localized in the ER [190]. There is also evidence that reticulon proteins play a role in clathrin-coated vesicular trafficking, by interacting with AP50, one of the AP2 adapter proteins [191]. The three chloroplast reticulons (Table 9) might then function as interactors with SNAREs and Rab GAPs in a COPII-related manner (Figures 6 and 7).

### Conclusion

The route taken by the chloroplast proteins entering from the cytosol and destined for the vesicles in our model is not clear, but two possibilities are considered here. The cargo proteins may leave the chloroplast envelope after passing the TOC-TIC complex into the stroma and then re-enter the chloroplast envelope to be transported to the thylakoid membrane. Alternatively, they may be arrested in the intermembrane space/inner envelope and then incorporated into vesicles. Regardless of the route, we suggest that the transmembrane-spanning proteins are directly bound by the coat proteins and the soluble cargo proteins are selected indirectly through transmembrane receptors attached to the membrane. Before any selection of cargo protein occurs, CPSAR1 is activated by a Sec12 GEF, which in turn recruits the Sec23–Sec24 complex. Sec24 then selects the cargo protein and the second layer of coating, consisting of Sec13, forms around the Sec23–Sec24, all causing membrane curvature. A vesicle buds after hydrolysis of CPSAR1, catalysed by its GAP Sec23. Putative Rabs may act in both the docking stage and in mediating fusion. The vesicles travel through the stroma and dock to the thylakoid membrane with the help of tethering factors, Rabs, SNAREs and SNARE-associated proteins. Finally, fusion occurs using v- and t-SNAREs with the assistance of SNARE-associated proteins, Rabs and reticulons and the cargo proteins are delivered to the thylakoid.

Most predicted budding components required for vesicle transport in chloroplasts are similar to COPII counterparts. However, the proteins involved in fusion are more similar to proteins of the late Golgi vesicle pathway, which operates between the Golgi and plasma membrane. A possible explanation for this could be that in cytosolic vesicle transport the Golgi is located between the ER and the final target site for processing proteins, but in chloroplasts there is no Golgi, no intermediate station. Thus, the chloroplast system may not require the components needed for Golgi fusion, and instead have homologues of the components required for late Golgi vesicle transport. Since the model of vesicle transport in chloroplasts presented in this paper is putative and based on tools not able to make 100% correct predictions, experimental verification is required to establish its true resemblance to the cytosolic vesicle transport system. Currently this is under investigation using *(inter alia*) e.g. spectroscopic analyses and protein-protein interactions. Several complications need to be addressed, e.g. the high frequency of dual-target components, the lack of evidence for chloroplast localization of previously predicted Sec proteins, missing participants or gaps in the Rab cycle, and the fact that vesicles are only visible only under certain conditions. Nevertheless, if predicted proteins are found to be true interactors our model would represent the first step towards understanding a new thylakoid protein targeting pathway, with novel implications for the assembly and maintenance of the photosynthetic machinery.

## Supporting Information

Figure S1 **A multiple sequence alignment including yeast Sec31, the best matches from the TAIR proteome (At1g18830 and At3g63460), and the best matches found by Andersson and Sandelius (2004) (At5g38560 and At2g45000).**
(RTF)

Figure S2 **A multiple sequence alignment including yeast Sec13, the best matches from the TAIR proteome (At3g01340 and At2g30050), and the best matches from the TAIR chloroplast proteome (At3g49660 and At2g43770).**
(RTF)

Figure S3 **A multiple sequence alignment including three putative chloroplast cargo receptor proteins (At1g72150, At4g09160, and At1g22530), and two other proteins (At1g30690, At3g51670) that have the same domains in the Arabidopsis proteome.**
(RTF)

Figure S4 **A multiple sequence alignment of the putative chloroplast AtCASP protein (At3g18480) with the best match in yeast (Coy1p) and human (CASP).**
(RTF)

Figure S5 **A multiple sequence alignment of the putative chloroplast SNAP protein (At5g61210) with the other two closely related SNAPs (At1g13890, At5g07788) in the Arabidopsis proteome.**
(RTF)

Figure S6 **A multiple sequence alignment of the putative chloroplast syntaxin protein (At5g16830) with the closely related yeast Pep12p and human syntaxin-7.**
(RTF)

Figure S7 **A multiple sequence alignment of the putative chloroplast SNARE associated Golgi protein (At1g22850) with the best hit found in yeast (Tvp38p) and in the Arabidopsis proteome (At2g02370).**
(RTF)

Figure S8 **A multiple sequence alignment of the putative chloroplast VAP protein (At4g05060) with the best hit found in yeast (Scs2p), human (VAPA) and the Arabidopsis proteome (At2g45140).**
(RTF)

Figure S9 **A multiple sequence alignment of the putative chloroplast AtRabA5e protein (At1g05810) with the best hit found in yeast (YPT31p), and human (Rab11A).**
(RTF)

Figure S10 **A multiple sequence alignment of the putative chloroplast AtRabF1 protein (At3g54840) with the best hit found in yeast (Vps12p) and human (Rab5B).**
(RTF)

Figure S11 **A multiple sequence alignment of the putative chloroplast AtRabB1c protein (At4g35860) with the best hit found in yeast (YPT1p) and human (Rab2A).**
(RTF)

Figure S12 **A multiple alignment of the putative chloroplast reticulon proteins (At2g20590, At4g28430, At5g58000) with the best hit found in yeast (RTN1) and the Arabidopsis proteome (At4g11220).**
(RTF)
