# Supplementary material for: New Putative Chloroplast Vesicle Transport Components and Cargo Proteins Revealed Using a Bioinformatics Approach: An Arabidopsis Model
Source: PLoS One. 2013 Apr 1;8(4):e59898. doi: 10.1371/journal.pone.0059898 (PMC3613420; doi:10.1371/journal.pone.0059898)
Supplement: Figure S1 — A multiple sequence alignment including yeast Sec31, the best matches from the TAIR proteome (At1g18830 and At3g63460), and the best matches found by Andersson and Sandelius (2004) (At5g38560 and At2g45000). (RTF) [file pone.0059898.s001.rtf]

Figure S1. A multiple sequence alignment including yeast Sec31, the best matches from the TAIR proteome (At1g18830 and At3g63460), and the best matches found by Andersson and Sandelius (2004) (At5g38560 and At2g45000). Identical residues are shown in black and conserved residues are shown in gray. Red color shows the WD repeat region, green color shows the protein kinase domain and the blue color shows the Ncp1 domain.


SEC31p       1 MVKLAEFSRTATFAWSHDKIPLLVSGTVSGTVDANFSTDSSLELWSLLAADSEKP---IA
At1g18830    1 MDCIKSIGRSAFVA-IAPESPFIAAGTMAGAVDLSFSSSANLEIFELDFQSNDRELKLVG
At3g63460    1 MACIKGVGRSASVA-LAPDAPYMAAGTMAGAVDLSFSSSANLEIFKLDFQSDDRDLPLVG
At5g38560    1 ------------------------------------------------------------
At2g45000    1 ------------------------------------------------------------


SEC31p      58 SLQVDSKFNDLDWSHNN--------KIIAGALDNGSLELYSTNEA----NNAINSMARFS
At1g18830   60 QCQSSERFNRLAWGSYGSGSD----GLIAGGLVDGNIGLWNPISS---ESGEIAHVRDLS
At3g63460   60 EIPSSERFNRLAWGRNGSGSEEFALGLIAGGLVDGNIDLWNPLSLIGSQPSENALVGHLS
At5g38560    1 ------------------------------------------------------------
At2g45000    1 ------------------------------------------------------------


SEC31p     106 NHSSSVKTVKFNAKQDNVLASGGNNGEIFIWDMNKCTESPSNYTPLTPGQSMSSVDEVIS
At1g18830  113 KHKGPVRGLEFNVKSPNQLASGADDGTVCIWDLANPSK-PSH-Y--LKGTGSYMQSEISS
At3g63460  120 VHKGPVRGLEFNAISSNLLASGADDGEICIWDLLKPSE-PSH-FPLLKGSGSATQGEISF
At5g38560    1 ------------------------------------------------------------
At2g45000    1 ------------------------------------------------------------


SEC31p     166 LAWNQSLAHVFASAGSSNFASIWDLKAKKEVIHLSYTSPNSGIKQQLSVVEWHPKNSTRV
At1g18830  169 LSWNKGFQHVLASTSHNGTTVIWDVNNEKIITDLK-----T--TVRCSVLQWDPDHFNQI
At3g63460  178 ISWNRKVQQILASTSYNGTTVIWDLRKQKPIINFA-----DSVRRRCSVLQWNPNVTTQI
At5g38560    1 ------------------------------------------------------------
At2g45000    1 ------------------------------------------------------------


SEC31p     226 ATATGSDNDPSILIWDLRNANTPLQTLNQGHQKGILSLDWCHQDEHLLLSSGRDNTVLLW
At1g18830  222 LVASDEDSSPNVKLLDIRYLQSPVRTF-VGHQRGVIAMEWCPSDSLYLLTCGKDNRTICW
At3g63460  233 MVASDDDSSPTLKLWDMRNIMSPVREF-TGHQRGVIAMEWCPSDSSYLLTCAKDNRTICW
At5g38560    1 ------------------------------------------------------------
At2g45000    1 ------------------------------------------------------------


SEC31p     286 NPESAEQLSQFPARGNWCFKTKFAPEAPDLFACASFDNKIEVQTLQNLTNTLDEQETETK
At1g18830  281 NTKTGKIVAELPTGQNWNFDVHWYPKMPGVISASSVDGKIGIYNLEGCSSYGTEN-----
At3g63460  292 DTNTAEIVAELPAGNNWNFDVHWYPKIPGVISASSFDGKIGIYNIEGCSRYGVEE-----
At5g38560    1 ------------------------------------------------------------
At2g45000    1 ------------------------------------------------------------


SEC31p     346 QQESETDFWNNVSREESKEKPTVFHLQAPTWYGEPSPAAHWAFGGKLVQITPDGKGVS--
At1g18830  336 ----QQHFLFH--------LLDADPLTAPKWWKRPAG-ASFGFGGKLISFNKNLPE----
At3g63460  347 ----NN--------------FGTAPLKAPKWYKRPVG-ASFGFGGKLVSCHARAPAKGTS
At5g38560    1 ------------------------------------------------------------
At2g45000    1 ------------------------------------------------------------


SEC31p     404 --ITNPKISGLESNTT-------LSEALKTK---DFKPLINQRLVKVIDDVNEEDWNLLE
At1g18830  379 --ASEVFLHSLATEKSLVNRISKFEAALENGEKTSLRGLCEKKTEEAESEEEKETWGLLK
At3g63460  388 SILSEVFLHSLVTEQSLVSRTSEFEAAIENGDMTSLRGLCEKKSEETESEEEKETWGLLK
At5g38560    1 ------------------------------------------------------------
At2g45000    1 ------------------------------------------------------------


SEC31p     452 KLSMDGTEE--FLKEALAFDNDE----------------------------SDAQDDANN
At1g18830  437 IMLEEDGNAKTKLRSHLGFSLPSEENDQTANEPHATCSSTNV------------------
At3g63460  448 IMFEEEGTSRTKLISHLGFTLPVAEKDQAVDGLSSDLNGIRLEDTAADALDLDDSNEAAA
At5g38560    1 ------------------------------------------------------------
At2g45000    1 ------------------------------------------------------------


SEC31p     482 EKEDDGEEFFQQIETN------------------F----------QPEGDFSLSGNIEQT
At1g18830  479 ------------------------------------EETQKVPEPEGEEEESSDPTFDDA
At3g63460  508 FAMDNGEDFFNNFPAKPDTPVSTSAKDFMPSDTDFSTKGEETQEMQEEEEESSDPVFDNA
At5g38560    1 ------------------------------------------------------------
At2g45000    1 ------------------------------------------------------------


SEC31p     514 ISKNLVSGNIKSAVKNSLENDLLMEAMVIALDSNNERLKESVKNAYFAKYGSKSSLSRIL
At1g18830  503 IQRSLIVGDYKEAVAQCFSANKMADALVIAHVGGT-ELWESTRDKYIRMSNA--PYMKVV
At3g63460  568 IQRALIVGDYKEAVDQCITANKMADALVIAHVGGT-ALWESTREKYLKTSSA--PYMKVV
At5g38560    1 ------------------------------------------------------------
At2g45000    1 ------------------------------------------------------------


SEC31p     574 YSISKREVDDLVENLDVSQWKFISKAIQNLYPNDIAQRNEMLIKLGDRLKENGHRQDSLT
At1g18830  560 SAMMNNDLMTYLHTRQPKSWKETLALICTFAEG--DEWISLCDALASNLMAAGFTLAATL
At3g63460  625 SAMVNNDLRSLIYTRSHKFWKETLALLCTFAQG--EQWTTLCDALASKLMAAGNTLAAVL
At5g38560    1 ------------------------------------------------------------
At2g45000    1 ------------------------------------------------------------


SEC31p     634 LYLAAGSLDKVASIWLSEFPDLEDKLKKDNKTIYEAHSECLTEFIERFTVFSNFINGSST
At1g18830  618 CYICAGNVDKTVDIWSMSLEKQSAG---------KSYAECVQDLMEKTLVLALTT-CNKR
At3g63460  683 CYICAGNVDRTVEIWSRSLANERDG---------RSYAELLQDLMEKTLVLALAT-GNKK
At5g38560    1 ------------------------------------------------------------
At2g45000    1 ---------------MSGFPFGQS------------------------NSVGGFSFGSSS


SEC31p     694 INNEQL-----IAKFLEFINLTTSTGNFELATEFLNSLPSDNEEVKTEKARVLIASGKSL
At1g18830  668 -VSASL-----RKLFESYAEILASQGLIATAMKFLKLLESGDFSPELSILR----DRISL
At3g63460  733 -FSASL-----CKLFESYAEILASQGLLTTAMKYLKVLDSGGLSPELSILR----DRISL
At5g38560    1 ------------------------------------------------------------
At2g45000   22 ATNSSSASSTTSPLSFSFNQ---SSNPSSTGFGFGSSVSS--------------------


SEC31p     749 PAQNPATATTSKAKYTNAKTN------KNVPVLPTPGMPS-TTSIPSMQAPFYGMTPGAS
At1g18830  718 YA-EPEAANTSASTN----------------------------TQPKI------------
At3g63460  783 SA-EPETNTTA-SGN----------------------------TQPQSTMPYNQ-EPTQA
At5g38560    1 ----------------------------------------------------------MS
At2g45000   59 ---TPASSTTPSFGFGASSTPSFGFGSSASSSTPSFGFGSSASVTPASTTPSFGFGTAAS


SEC31p     802 ANALPPKPYVPATTTSAPVHTEGKYAPP-SQPSMASPF--VNKTNSSTR-----------
At1g18830  737 --------------------------------------------SNPYQ-----------
At3g63460  812 QPNVLANPYDNQYQ---QPYTDSYYVPQVSHPPMQQPTMFMPHQAQPAP-----------
At5g38560    3 LVPPLPIL-SPPSSN------SS------TTAPP--P-----------------LQTQPT
At2g45000  116 SSAPAPSLFGSSTTN------AS------SAAPGSSPFGFVTSSASSTATPSSSLFGAPA


SEC31p     848 ----LNSFAPPPNPYATATVPATNVSTTSIPQNTFAPIQPGMPIMGDYNAQSSSIPSQPP
At1g18830  742 ----EKSFTPAPLSNAQP-----------SRSITFFPL-------------------NPP
At3g63460  858 ----QPSFTPAPTSNAQP-----------SMRTTFVPS-------------------TPP
At5g38560   31 TPSAPPPVTPPPS-PPQSPPPVVS--SSPPPPVVSSPPPSS------------SPPPSPP
At2g45000  164 SSAATPSSSPFGAAPASGSTPLFG--SSP--SLFSAPSSASASNSSLFGASSSAATSTSP


SEC31p     904 I------NAVSGQTPHLNRKANDGWNDLPLKVKE-KPSRAKAVSVAPPNILS--------
At1g18830  768 R------EL----------KNADQYQQPTMDY----------------------------
At3g63460  884 -------AL----------KNADQYQQPTMSS----------------------------
At5g38560   76 VITSPPPTVASSPPPPV-------------VIASPPPSTPATTPPAPPQTVSPPPPPDAS
At2g45000  220 LFGAPSSAT--GATPSF-------------SVASSAPGS--------------------S


SEC31p     949 --------------------------------------------TPTPLNGIPANAASTM
At1g18830  784 ----------------------------------------------HSFNR-SAGPAYNA
At3g63460  899 ----------------------------------------------HSFTG-PSNNAYPV
At5g38560  123 PSPPAPTTTNPPPKPSPSPPGETPSPPGETPSPPKPSPS---TPTPT--------TTTSP
At2g45000  245 SSIFGATGSSPSFSVASSASGSSPSIFGATGSSPFFGSSSSAGSTPSLFASSSSGATTSS


SEC31p     965 PPPPLSRAPSS-V----SMVSPPPLHKNSRVPSLVATSESPRASISNPYAPPQ---SSQQ
At1g18830  797 PPGPGSYRSI--H----SQV----------------------------------------
At3g63460  912 PPGPGQYAPSG-P----SQL----------------------------------------
At5g38560  172 PPPPATSASP--PSSNP------------TDPSTLAPP-----PTPLPVVPRE---KPIA
At2g45000  305 PSPFGVSTFNSSSTSNTSNASASPFSAS-TGFSFLKSTASSTTSSTTPSAPPQTASSSSS


SEC31p    1017 FPIGTISTANQTSNT------AQVASSNPYA-------------PPPQQRVATPLSGGVP
At1g18830  811 ---------------------------GPYI-------------NSKIPQTVAPPVRPMT
At3g63460  927 ---------------------------GQYP-------------NPKMPQVVAPAAGPIG
At5g38560  210 KPTGPASNNGNNTLPSSSPGKSEVGTGGIVAIGVIV--------------------G-LV
At2g45000  364 FSFGTSANSGFNLSTGSSAAPASSTSGAVFSIATTTTTSSSTPAATSAPASSAPAST-MA


SEC31p    1058 PAPLPKAS-----NPYAP------------------TATTQPNGSSYPPTGPYTNNHTM-
At1g18830  831 P------------------------------------------------------THQV-
At3g63460  947 FTPMAT---------------------------------PGVAPRSVQPASPPTQQAAA-
At5g38560  249 FLSLFV------------------MGVWFT-RKRKRKDPGTFVGYTMPPSAYS----SPQ
At2g45000  423 FPSFGVTSSATNTTPASSAATFSTTGFGLASSTPATGSTNSFTGFAVPKTSTPASSSQPQ


SEC31p    1094 TSPPPVFNKPPTGPPPISMKKRSNKLAS------------------------------IE
At1g18830  836 A-----------------------------------------------------------
At3g63460  973 Q-----------------------------------------------------------
At5g38560  286 GSDVVLFNSRSSAPPKMRSHSGSDYMYASSDSGMVSNQRSWFSYDELSQVTSGFSEKNLL
At2g45000  483 TTS------------------------------------PAFSFSLPSSTSTTAPA----


SEC31p    1124 QNPSQGATYPPTLSSSASPLQPSQPPTLASQVNTSAENVSHEIPADQQPIVDFLKEE---
At1g18830  837 ----------------VQPEPVAPPPTVQ-------TADTSNVPAHQKPIVASLTRLFKE
At3g63460  974 ----------------AAPAPATPPPTVQ-------TADTSNVPAHQKPVIATLTRLFNE
At5g38560  346 GEGGFGCVYKGVLSDGREVA--------VKQLKIGGSQGEREFK----AEVEIISRVHHR
At2g45000  503 -------------TSSATTT--------QTTLVVPSSSGTSTAV----APV---------


SEC31p    1181 ----LA---------------------------------------RVTPLTPKEYSKQLK
At1g18830  874 TFEPLR---------------------------------------GYSRDTPAKKREAED
At3g63460 1011 TSEALG---------------------------------------GARANTT-KKREI-E
At5g38560  394 HLVTLVGYCISEQHRLLVYDYVPNNTLHYHLHAPGRPVMTWETRVRVAAGAARGIAYLHE
At2g45000  529 -----AG--------------SPKLPSEITGKTVEEIIKEWNTELQERTGRFRKQANAIA


SEC31p    1198 DCDKRLKILFYHLEKQDLLTQPTIDCLHD------LVALMKEKKYK--EAMVIHANIATN
At1g18830  895 NCSRKLGALFSKLNNGDISKN-AAEKLTQ------LCQALDKRDFG--AALKIQGLMTST
At3g63460 1030 DNSRKLGALFVKLNSGDISKN-AADKLAQ------LCQALDNNDFS--TALQIQVLLTTS
At5g38560  454 DCHPRI--IHRDIKSSNILLDNSFEALVADFGLAKIAQELDLNTHVSTRVMGTFGYMAPE
At2g45000  570 EWDKRI--LQ----NRDVLLRLEIEVAKVVETQSSLERQLELIETHQQEVDKALQSMEE-


SEC31p    1250 HAQ-----E-GGNWLTGV------------------------------------------
At1g18830  946 EWD-----E-CSSWLPTL------------------------------------------
At3g63460 1081 EWD-----E-CNFWLATL------------------------------------------
At5g38560  512 YATSGKLSEKADVYSYGVILLELITGRKPVDTSQPLGDESLVEWARPLLGQAIENEEFDE
At2g45000  623 --------EAERI----------------------------------------YNDERKS


SEC31p    1262 ---------------KRLIGIAEATLN---------------------------------
At1g18830  958 ---------------KKMIVTGRQNVR---------------------------------
At3g63460 1093 ---------------KRMMVKARQNVR---------------------------------
At5g38560  572 LVDPRLGKNFIPGEMFRMVEAAAACVRHSAAKRPKMSQVVRALDT-LEEATDITNGMRPG
At2g45000  635 LLDDEAAS--TRDAMYEQSELVERELEHMTEQ---IRSIIQSVNANQGGELEAIDGMSPL


SEC31p         ---------------------------------------------------------
At1g18830      ---------------------------------------------------------
At3g63460      ---------------------------------------------------------
At5g38560  631 QSQVFDSRQQSAQIRMFQRMAFGSQDYSSDFFDRSQSHSSWGSRDQSRFV-P-----
At2g45000  690 DVVVRILN---NQLSSLMWID----EKAEEFSSRIQKIALQGSGGDRELMAPKHWMS
