# Supplementary material for: New Putative Chloroplast Vesicle Transport Components and Cargo Proteins Revealed Using a Bioinformatics Approach: An Arabidopsis Model
Source: PLoS One. 2013 Apr 1;8(4):e59898. doi: 10.1371/journal.pone.0059898 (PMC3613420; doi:10.1371/journal.pone.0059898)
Supplement: Figure S2 — A multiple sequence alignment including yeast Sec13, the best matches from the TAIR proteome (At3g01340 and At2g30050), and the best matches from the TAIR chloroplast proteome (At3g49660 and At2g43770). (RTF) [file pone.0059898.s002.rtf]

Figure S2. A multiple sequence alignment including yeast Sec13, the best matches from the TAIR proteome (At3g01340 and At2g30050), and the best matches from the TAIR chloroplast proteome (At3g49660 and At2g43770). Identical residues are shown in black and conserved residues are shown in gray. Red color shows the WD repeat region.

Sec13p       1 --------------------------------------------MVVIANAHNELIHDAV
At3g01340    1 ------------------------------------------MPPQKIETGHSDTIHDVV
At2g30050    1 ------------------------------------------MPGQKIETGHEDIVHDVQ
At3g49660    1 ------------------MAEEIPATASFTPY-----------VHSQTLTSHNRAVSSVK
At2g43770    1 MEIMSRENETALSGPRPMEWSTVPHSASQGPGPNGKNRTSSLEAPIMLLSGHPSAVYTMK


Sec13p      17 LDYYGKRLATCSSDKTIKIFEVEGE---THKLIDTLTGHEGPVWRVDWAHPKFGTILASC
At3g01340   19 MDYYGKRVATASSDCTIKITGVSNSG--GSQHLATLTGHRGPVWQVAWAHPKFGSLLASC
At2g30050   19 MDYYGKRIATASSDCTIKITGVSNNG--GSQQLATLTGHRGPVWEVAWAHPKYGSILASC
At3g49660   32 FSSDGRLLASASADKTIRTYTINTINDPIAEPVQEFTGHENGISDVAFSS--DARFIVSA
At2g43770   61 FNPAGTLIASGSHDREIFLWRVHGD----CKNFMVLKGHKNAILDLHWTS--DGSQIVSA


Sec13p      74 SYDGKVLIWKEEN-GRWSQIAVHAVHSASVNSVQWAPHEY-GPLLLVASSDGKVSVVEFK
At3g01340   77 SYDGQIILWKEGNQNQWTQAHVFTDHKVSVNSIAWAPHEL-GLSLACGASDGNISVFSAR
At2g30050   77 SYDGQVILWKEGNQNQWTQDHVFTDHKSSVNSIAWAPHDI-GLSLACGSSDGNISVFTAR
At3g49660   90 SDDKTLKLWDVETGSL---IKTLIGHTNYA-FC--VNFNPQSNMIVSGSFDETVRIWDVT
At2g43770  115 SPDKTVRAWDVETGKQ---IKKMAEHSSFVNSC--CPTRRGPPLIISGSDDGTAKLWDMR


Sec13p     132 ENGT-TSPIIIDAHAIGVNSASWAPATIEEDGEHNGT-KESRKFVTGGADNLVKIWKYNS
At3g01340  136 ADGGWDTTKIDQAHPVGVTSVSWAPATEPGALVSSGMIDPVYKLASGGCDSTVKVWKFSN
At2g30050  136 ADGGWDTSRIDQAHPVGVTSVSWAPATAPGALVSSGLLDPVYKLASGGCDNTVKVWKLAN
At3g49660  144 TGKCLKV---LPAHSDPVTAVDF--------------NRDGSLIVSSSYDGLCRIWDSGT
At2g43770  170 QRGAIQT---FPDKY-QITAVSF--------------SDAADKIFTGGVDNDVKVWDLRK


Sec13p     190 DAQTYVLESTLEGHSDWVRDVAWSPTVLL-RSYLASVSQDRTCIIWTQDNEQGPWKKTL-
At3g01340  196 GSWKMDCFPALNKHTDWVRDVAWAPNLGLPKSTIASGSEDGKVIIWTIGKEGEQWEGTV-
At2g30050  196 GSWKMDCFPALQKHTDWVRDVAWAPNLGLPKSTIASGSQDGKVIIWTVGKEGEQWEGKV-
At3g49660  187 GHCVKTLIDDE---NPPVSFVRFSPN----GKFILVGTLDNTLRLWNISSA----KFLKT
At2g43770  212 GEATMTLEGH----QDTITGMSLSPD----GSYLLTNGMDNKLCVWDMRPYAPQNRCVKI


Sec13p     248 --LKEEKFPDVLWRASWSLSGNVLALSGGDNKVTLWKENLE-------GKWEPAGEV--H
At3g01340  255 --LKD--FKTPVWRVSWSLTGNLLAVSDGNNNVTVWKESVD-------GEWEQVTVV--E
At2g30050  255 --LKD--FMTPVWRVSWSLTGNLLAVSDGNNNVTVWKEAVD-------GEWEQVTAV--E
At3g49660  236 YTGHVNAQ-YCISSAFSVTNGKRIVSGSEDNCVHMWELNSKKLLQKLEGHTETVMNVACH
At2g43770  264 FEGHQHNFEKNLLKCSWSPDGTKVTAGSSDRMVHIWDTTSRRTIYKLPGHTGSVNECVFH


Sec13p     297 Q----------------------
At3g01340  302 P----------------------
At2g30050  302 P----------------------
At3g49660  295 PTENLIASGSLDKTVRIWTQKKE
At2g43770  324 PTEPIIGSCSSDKNIYLGEI---
