# Supplementary material for: New Putative Chloroplast Vesicle Transport Components and Cargo Proteins Revealed Using a Bioinformatics Approach: An Arabidopsis Model
Source: PLoS One. 2013 Apr 1;8(4):e59898. doi: 10.1371/journal.pone.0059898 (PMC3613420; doi:10.1371/journal.pone.0059898)
Supplement: Figure S3 — A multiple sequence alignment including three putative chloroplast cargo receptor proteins (At1g72150, At4g09160, and At1g22530), and two other proteins (At1g30690, At3g51670) that have the same domains in the Arabidopsis proteome. (RTF) [file pone.0059898.s003.rtf]

Figure S3. A multiple sequence alignment including three putative chloroplast cargo receptor proteins (At1g72150, At4g09160, and At1g22530), and two other proteins (At1g30690, At3g51670) that have the same domains in the Arabidopsis proteome. Identical residues are shown in black and conserved residues are shown in gray. Red color shows the CRAL_TRIO domain, green color shows the GOLD domain and orange color shows the overlapping sequence between the two domains.

At1g72150    1 MAQEEVQKSADVAAAPVVKE-KPITDKEVTIPTPVAEK--------EEVAAP--------
At4g09160    1 MSQDSAT----TTPPPPLTSDVSMPSGEEDEPKHVTSEEEAPVTSETNLKLPLMPELEES
At1g22530    1 MAQEEIQKPTA--SVPVVKEETPAPVKEVEVPVT--TE--------KAVAAPA-PEAT--
At1g30690    1 ------------------------------------------------------------
At3g51670    1 ------------------------------------------------------------


At1g72150   44 -----VSDEKAVPEKEVTPEKEA-PAAE-AEKSVSVKEEETVVVAEKVVVLTAEEVQK--
At4g09160   57 NHTAEVVSEKVTPETMTLESEGLNHAAEDSEQTHEVTPETETAKL-EVLNHTAEDSEQTH
At1g22530   46 --EEKVVSEVAVPETEVTAVKEE-EVAT-GKEILQ----SESFKEEGYLASELQEAEK--
At1g30690    1 ---------------------------------------------------------MTA
At3g51670    1 ------------------------------------------------------------


At1g72150   95 KALEEFK----ELVREALNKREFTAPVTP----VKEEKTEEKKTEEETKE--EE-KTEEK
At4g09160  116 EVTPEKETVKSEFLNHVAEDSEQTHEVTPETETVKSE-VLN-HAAEDSEQPRGVTPTPET
At1g22530   96 NALAELK----ELVREALNKREFTAPPPPPA-PVKEEKVEEKKTEETEEKKEEV-KTEEK
At1g30690    4 EVKVEEKQVESEVV--------IAPAVVPEETTVKAV-VEETKVEEDESKPEGVEKSASF
At3g51670    1 ------------------------------------------------------------


At1g72150  144 K----EETTT------------EV---------------------------KVEEEKPAV
At4g09160  174 E--------------------------TSEADTS-LL-VTSETEEPNHAAEDYSETEPSQ
At1g22530  150 S----LEAETKEEEKSAAPATVETKKEEILAAPAPIVAETKKEETPV--APAPVETKPAA
At1g30690   55 KEESDFFADLKESEKKAL-SDLKSKLEEAIVDNT-LL-KTKKKES-----------SP--
At3g51670    1 ------------------------------------------------------------


At1g72150  161 PAAEEEKSSE---AA---------------------PVETKSE--EK---------PEE-
At4g09160  206 KLMLEQRRK-YM--------------EVEDWTEPELPDEAVLE---AAASVPEPKQPEPQ
At1g22530  204 PVVAETKKEEILPAAPVTTETKVEEKVVPVETTPAAPVTTETKEEEKAAPVTTETKEEEK
At1g30690   99 --MKEKKE---------------------EVVKPEAEVEKKKEEA-AEEKVEEEKKSEAV
At3g51670    1 -----------------------------------------MD---A--SLSPFDHQKTQ


At1g72150  185 ----------KAE---------------------------------VTTEK-ASSAEEDG
At4g09160  248 -----T-PPPPPSTTTST-VASRSLAEMMN----REEAEVEEK-QKIQIPRSLGSFKEET
At1g22530  264 AAPGETKKEEKATASTQVKRASKFIKDIFVSVTTSEKKKEEEKPAVVTIEKAFAADQEEE
At1g30690  135 ----VTEEAPKAETVEAV-VT----EEIIP----KEEV-------TTVVEKVEEETKEEE
At3g51670   15 -----N-TEPKKSFIT------------------------------SLITLRSNNIKEDT


At1g72150  201 TKTVEAIEESIVSVSPPESAVAPVVVETVAVAEAEPVEPEEVSIWGVPLLQ---DERSDV
At4g09160  296 NKISDLSETELN------------ALQELRHLLQVSQDSSKTSIWGVPLLK---DDRTDV
At1g22530  324 TKTVEAVEESIVSITLP--------------ETAAYVEPEEVSIWGIPLLE---DERSDV
At1g30690  175 KKTEDVVTEEVK------------A-ETIEVEDEDESVDKDIELWGVPLLPSKGAESTDV
At3g51670   39 YFVSELKPTEQK------------SLQELKEKLSAS-SSKASSMWGVSLLG--GDDKADV


At1g72150  258 ILTKFLRARDFKVKEALTMLKNTVQWRKENKIDELVESGEEV-SEFEKMVFAHGVDKEGH
At4g09160  341 VLLKFLRARDFKPQEAYSMLNKTLQWRIDFNIEELLDENLGD--DLDKVVFMQGQDKENH
At1g22530  367 ILLKFLRARDFKVKEAFTMLKNTVQWRKENKIDDLVSEDLEG-SEFEKLVFTHGVDKQGH
At1g30690  222 ILLKFLRARDFKVNEAFEMLKKTLKWRKQNKIDSILGEEFGE--DLATAAYMNGVDRESH
At3g51670   84 ILLKFLRARDFKVADSLRMLEKCLEWREEFKAEKLTEEDLGFKDLEGKVAYMRGYDKEGH


At1g72150  317 VVIYSSYGEFQNKELF----SDKEKLNKFLSWRIQLQEKCVRAIDFSNPEAKSSFVFVSD
At4g09160  399 PVCYNVYGEFQNKDLYQKTFSDEEKRERFLRWRIQFLEKSIRNLDFV-AGGVSTICQVND
At1g22530  426 VVIYSSYGEFQNKEIF----SDKEKLSKFLKWRIQFQEKCVRSLDFS-PEAKSSFVFVSD
At1g30690  280 PVCYNVHSE----ELY-QTIGSEKNREKFLRWRFQLMEKGIQKLNLK-PGGVTSLLQIHD
At3g51670  144 PVCYNAYGVFKEKEMYERVFGDEEKLNKFLRWRVQVLERGVKMLHFK-PGGVNSIIQVTD


At1g72150  373 FRNAPGLGKRALWQFIRRAVKQFEDNYPEFAAKELFINVPWWYIPYYKTFGSIITSPRTR
At4g09160  458 LKNSPGPGKTELRLATKQALHLLQDNYPEFVSKQIFINVPWWYLAFYRIISPFM-SQRSK
At1g22530  481 FRNAPGLGQRALWQFIKRAVKQFEDNYPEFVAKELFINVPWWYIPYYKTFGSIITSPRTR
At1g30690  334 LKNAPGVSRTEIWVGIKKVIETLQDNYPEFVSRNIFINVPFWFYAMRAVLSPFL-TQRTK
At3g51670  203 LKDMP---KRELRVASNQILSLFQDNYPELVATKIFINVPWYFSVIYSMFSPFL-TQRTK


At1g72150  433 SKMVLAGPSKSADTIFKYIAPEQVPVKYGGLSKD-----TPLTE-ETITEAIVKPAANYT
At4g09160  517 SKLVFAGPSRSAETLLKYISPEHVPVQYGGLSVDNCECNSDFTHDDIATEITVKPTTKQT
At1g22530  541 SKMVLSGPSKSAETIFKYVAPEVVPVKYGGLSKD-----SPFTVEDGVTEAVVKSTSKYT
At1g30690  393 SKFVVARPAKVRETLLKYIPADELPVQYGGFKTVDDTEFS----NETVSEVVVKPGSSET
At3g51670  259 SKFVMSKEGNAAETLYKFIRPEDIPVQYGGLSRPTDSQNGP---PKPASEFSIKGGEKVN


At1g72150  487 IELP-ASEACTLSWELRVLGADVSYGAQFEPTTEGSYAVIVSKTRKIGSTDEPVITDSFK
At4g09160  577 VEII-VYEKCTIVWEIRVVGWEVSYGAEFVPENKEGYTVIIQKPRKMTAKNELVVSHSFK
At1g22530  596 IDLP-ATEGSTLSWELRVLGADVSYGAQFEPSNEASYTVIVSKNRKVGLTDEPVITDSFK
At1g30690  449 IEIPAPETEGTLVWDIAVLGWEVNYKEEFVPTEEGAYTVIVQKVKKMGANEGP-IRNSFK
At3g51670  316 IQIEGIEGGATITWDIVVGGWDLEYSAEFVPNAEESYAIVVEKPKKMKATDEAV-CNSFT


At1g72150  546 VGEPGKIVITIDNQTSKKK-KVLYRFKTQ------
At4g09160  636 VGEVGRILLTVDNPTSTKK-MLIYRFKVKPLACE-
At1g22530  655 ASEAGKVVITIDNQTFKKK-KVLYRSKTQA-----
At1g30690  508 NSQAGKIVLTVDNVSGKKK-KVLYRYRTKTESSS-
At3g51670  375 TVEAGKLILSVDNTLSRKKKVAAYRYTVRKSTTTV
