# Supplementary material for: New Putative Chloroplast Vesicle Transport Components and Cargo Proteins Revealed Using a Bioinformatics Approach: An Arabidopsis Model
Source: PLoS One. 2013 Apr 1;8(4):e59898. doi: 10.1371/journal.pone.0059898 (PMC3613420; doi:10.1371/journal.pone.0059898)
Supplement: Figure S4 — A multiple sequence alignment of the putative chloroplast AtCASP protein (At3g18480) with the best match in yeast (Coy1p) and human (CASP). (RTF) [file pone.0059898.s004.rtf]

Figure S4. A multiple sequence alignment of the putative chloroplast AtCASP protein (At3g18480) with the best match in yeast (Coy1p) and human (CASP). Identical residues are shown in black and conserved residues are shown in gray. Red color shows the CASP domain.

Coy1p        1 --------------------MDTSVYSHALDIWAKADLTNLQRELDADVIEIKDKETLSL
CASP         1 ---------------------MAANVGSMFQYWKRFDLQQLQRELDATATVLANRQDESE
At3g18480    1 MEVSQDGSERDKTPPPSSSSSSSSPIPVVTNFWKEFDLEKEKSLLDEQGLRIAENQENSQ


Coy1p       41 NSRKSLATETKKFKKLEPEEKLNNVNKIIKQYQREIDNLTQRSKFSEKVLFDVYEKLSEA
CASP        40 QSRKRLIEQSREFKKNTPEDLRKQVAPLLKSFQGEIDALSKRSKEAEAAFLNVYKRLIDV
At3g18480   61 KNRRKLAESTRDFKKASPENKLSMFNSLLKGYQEEVDNITKRAKFGENAFLNIYQKLYEA


Coy1p      101 PDPQPLLQSSLEKLGKIDDSKELKEKISYLEDKLAKYA-----------DYETLKSRLLD
CASP       100 PDPVPALDLGQQLQLKVQRLHDIETENQKLRETLEEYNKEFAEVKNQEVTIKALKEKIRE
At3g18480  121 PDPFPALASIAE---QDRKLSEVESENRKMKVELEEFRTEATHLKNQQATIRRLEERNRQ


Coy1p      150 LEQSSAKTLAKRLTAKTQEINSTWEEKGRNWKEREADLLKQLTNVQE----QNKALEAKI
CASP       160 YEQTLKNQAETIALEKEQKLQNDFAEKERKLQETQMSTTSKLEEAEHKVQSLQTALEKTR
At3g18480  178 LEQQMEEKIKEVVEIKQRNLAEENQKTMELLKDREQALQDQLRQAKDSVSTMQKLHELAQ


Coy1p      206 SKNIDIEGNGNEDGDQENNQKEVSTRIAEYNLVTQELETTQARIYQLEKRNEELSGALAK
CASP       220 TELFDLKTKYD---------EETTAKADEIEMIMTDLERANQRAEVAQREAETLREQLSS
At3g18480  238 NQLFELRAQSD---------EETAGKQSEVSLLMDEVERAQTRLLTLEREKGHLRSQLQT


Coy1p      266 ATS--------------------EAEKETELHAKELKLNQLESENALLSASYEQERKSTS
CASP       271 ANHSLQLASQIQKAPDVAIEVLTRSSLEVELAAKEREIAQLVEDVQRLQASLTKLRENSA
At3g18480  289 ANEDTDNKKS----D----NIDSNSMLENSLTAKEKIISELNMEIHNVETALANERESHV


Coy1p      306 HAINELKEQLNSVVAESESYKSELETVRRKLNNYSDYNKIKEELSALKKIEFGVNEDDSD
CASP       331 SQISQLEQQLSAKNSTL----KQLEEKLKGQA---DYEEVKKELNILKSMEFAPSE----
At3g18480  341 AEIKKLNSLLNKKDTII----EEMKKELQERPSAKLVDDLRKKVKILQAVGYNSIEAEDW


Coy1p      366 NDIRSEDKNDNTFESSLLSANKKLQATLAEYRSKSTAQEEERNELKKSVDQLKQQIATLK
CASP       380 -G-AGTQDAAKPLEVLLLEKNRSLQSENAALRISNSDLSGRCAELQVRITEAVATATEQR
At3g18480  397 DA-ATTGEEMSKMESLLLDKNRKMEHEVTQLKVQLSEKASLLEKAEAKGEELTAKVNEQQ


Coy1p      426 EANEKLETDLEKVENVS------PHFNETASMMSGVTRQMNNRTSHKMSPTSSIIGIPED
CASP       438 ELIARLEQDLSIIQSIQRPDAEGAAEHRLEKIPEPIK----EATALFYGPAAPASGALP-
At3g18480  456 RLIQKLEDDIL--KGYGSKERKGALFDEWEFSEAGVA----EQS----EP-MDQKHVPS-


Coy1p      480 GELSGNQSTILPIVTKQRDRFRSRNMDLEKQLRQGNSEKGKLKLEISKLKGDNTKLYERI
CASP       493 ---EGQVDSLLSIISSQRERFRARNQELEAENRLAQHTLQALQSELDSLRADNIKLFEKI
At3g18480  504 ---EQDQSSMLKVICSQRDRFRARLRETEEEIRRLKEKIGFLTDELEKTKADNVKLYGKI


Coy1p      540 RYLQSYNNNNAPVNQS---------TERIDVESQYSRVYDESLHPMANFRQNELNHYKNK
CASP       550 KFLQSYPGRGS---------------GSDDTELRYSSQYEERLDPFSSFSKRERQ-RKYL
At3g18480  561 RYVQDYNHDKVVSRGSKKYVEDLESGFSSDVESKYKKIYEDDINPFAAFSKKERE-QRIK


Coy1p      591 KLSALEKLFSSFAKVILQNKMTRMVFLFYCIGLHGLVFMMSMYVINISGYMTPEVGIVQS
CASP       594 SLSPWDKATLSMGRLVLSNKMARTIGFFYTLFLHCLVFLV-LYKLAWSESMERDCATFC-
At3g18480  620 DLGIRDRITLSSGRFLLGNKYARTFAFFYTIGLHVLVFTC-LYRMSAYSYLSHGAEETLM


Coy1p      651 AKSSSNLNGGLGGAEKVAAGVGSVHGINR
CASP       652 AKKFADHLHKFHENDNGAA-AGDLWQ---
At3g18480  679 TEATTNLPHGL------------------
