# Supplementary material for: New Putative Chloroplast Vesicle Transport Components and Cargo Proteins Revealed Using a Bioinformatics Approach: An Arabidopsis Model
Source: PLoS One. 2013 Apr 1;8(4):e59898. doi: 10.1371/journal.pone.0059898 (PMC3613420; doi:10.1371/journal.pone.0059898)
Supplement: Figure S5 — A multiple sequence alignment of the putative chloroplast SNAP protein (At5g61210) with the other two closely related SNAPs (At1g13890, At5g07788) in the Arabidopsis proteome. (RTF) [file pone.0059898.s005.rtf]

Figure S5. A multiple sequence alignment of the putative chloroplast SNAP protein (At5g61210) with the other two closely related SNAPs (At1g13890, At5g07788) in the Arabidopsis proteome. Identical residues are shown in black and conserved residues are shown in gray. Red color shows the t-SNARE domain.

At1g13890    1 MFGFFKSPGNNKLPN--------------ESSNNKGGTITAGRRTSSEPILIT--P----
At5g07880    1 ----------------MAPKNSSWNPFDDEKEAAKSFSLNP----------------FDD
At5g61210    1 MFGLRKSPANLPKHNSVDLKSSKPNPFDSDDESDNKHTLNPSKRTTSEPSLADMTNPFGG


At1g13890   41 ---------------DFDDDDKYKNGFNDSGGLQSQTTEELEKYAVYKAEETTKGVNNCL
At5g07880   29 DDD------------DKEVEKRFTSSLKPSGGKENQTVQELESYAVYNSEETTKTVQGCL
At5g61210   61 ERVQKGDSSSSKQSLFSNSKYQYKNNFRDSGGIENQSVQELEGYAVYKAEETTKSVQGCL


At1g13890   86 KIAEDIRSDGARTLEMLHQQGEQINRTHEMAVDMDKDLSRGEKLLNNLGGMFSKPWKPKK
At5g07880   77 KVAEEIRCDASKTLVMLNEQGDQITRTHQKTVDLDHHLSRGEKILGRLGGVFSRTWKPKK
At5g61210  121 KVAEDIRSDATRTLVMLHDQGEQITRTHHKAVEIDHDLSRGEKLLGSLGGMFSKTWKPKK


At1g13890  146 TKNITGPMITPDKPSKKSENHKEEREKLGLGAKGR--SSSQPALDQPTNALQKVEQEKAK
At5g07880  137 SRSITGPVITKGDSPKRKVIDLKTREKLGLNPSLKPKS---KTLPEAVDAYQKTQI--AK
At5g61210  181 TRPINGPVVTRDDSPTRRVNHLEKREKLGLNSAPRGQSRTREPLPESADAYQRVEMEKAK


At1g13890  204 QDDGLSDLSDILGDLKSMAVDMGSEIDKQNKALDHLGDDVDELNSRVQGANQRARHLLSK
At5g07880  192 QDEALTDLSALLGELKNMAVDMGTAIERQTNELDHLQDNADELNYRVKQSNQRARYLLRK
At5g61210  241 QDDGLSDLSDILGELKNMAVDMGSEIEKQNKGLDHLHDDVDELNFRVQQSNQRGRRLLGK
