# Supplementary material for: New Putative Chloroplast Vesicle Transport Components and Cargo Proteins Revealed Using a Bioinformatics Approach: An Arabidopsis Model
Source: PLoS One. 2013 Apr 1;8(4):e59898. doi: 10.1371/journal.pone.0059898 (PMC3613420; doi:10.1371/journal.pone.0059898)
Supplement: Figure S6 — A multiple sequence alignment of the putative chloroplast syntaxin protein (At5g16830) with the closely related yeast Pep12p and human syntaxin-7. (RTF) [file pone.0059898.s006.rtf]

Figure S6. A multiple sequence alignment of the putative chloroplast syntaxin protein (At5g16830) with the closely related yeast Pep12p and human syntaxin-7. Identical residues are shown in black and conserved residues are shown in gray. Red color shows the t-SNARE domain.

Pep12p        1 ------------MSEDEFFGGDNEAVWNGSRFSDSPEFQTLKEEVAAELFEINGQISTLQ
syntaxin-7    1 ---------------------MSYTPGV------GGDPAQLAQRISSNIQKITQCSVEIQ
At5g16830     1 MSFQDLEAGTRSPAPNRFTGGRQQRPSS------RGDPSQ---EVAAGIFRISTAVNSFF


Pep12p       49 QFTATLKSFIDRGDVSAKVVERINKRSVAKIEEIGGLIKKVNTSVKKMDAI-EEASLDKT
syntaxin-7   34 RTLNQLGTPQDSPELRQQ----LQQ----KQQYTNQLAKETDKYIKEFGSLPTTPSEQRQ
At5g16830    52 RLVNSIGTPKDTLELRDK----LQK----TRLQISELVKNTSAKLKEASEADLHGSASQI


Pep12p      108 QIIAREKLVRDVSYSFQEFQGIQRQFTQVMKQVNERAKESLEASEMANDAALLDEEQRQN
syntaxin-7   86 RKIQKDRLVAEFTTSLTNFQKVQRQAAEREKEFVARVRASSRVSGSF---PE-------D
At5g16830   104 KKIADAKLAKDFQSVLKEFQKAQRLAAEREITYTPVVTKEIPTSYNA---PE-------L


Pep12p      168 SSKSTRIPGSQ-------IVIERDPINNEEFAYQQNLIEQRDQEISNIERGITELNEVFK
syntaxin-7  136 SSKERNL-----VSWESQTQPQVQVQDEEITEDDLRLIHERESSIRQLEADIMDINEIFK
At5g16830   154 DTESLRISQQQALLLQSRR-QEVVFLDNEIT-FNEAIIEEREQGIREIEDQIRDVNGMFK


Pep12p      221 DLGSVVQQQGVLVDNIEANIYTTSDNTQLASDELRKAMRYQKRTSRWRVYLLIVLLVMLL
syntaxin-7  191 DLGMMIHEQGDVIDSIEANVENAEVHVQQANQQLSRAADYQRKSRKTLCIIILILVIGVA
At5g16830   212 DLALMVNHQGNIVDDISSNLDNSHAATTQATVQLRKAAKTQRSNSSLTCLLILIFGIVLL


Pep12p      281 FIFLIM-KL--
syntaxin-7  251 IISLIIWGLNH
At5g16830   272 IVIIVVLV---
