# Supplementary material for: New Putative Chloroplast Vesicle Transport Components and Cargo Proteins Revealed Using a Bioinformatics Approach: An Arabidopsis Model
Source: PLoS One. 2013 Apr 1;8(4):e59898. doi: 10.1371/journal.pone.0059898 (PMC3613420; doi:10.1371/journal.pone.0059898)
Supplement: Figure S7 — A multiple sequence alignment of the putative chloroplast SNARE associated Golgi protein (At1g22850) with the best hit found in yeast (Tvp38p) and in the Arabidopsis proteome (At2g02370). (RTF) [file pone.0059898.s007.rtf]

Figure S7. A multiple sequence alignment of the putative chloroplast SNARE associated Golgi protein (At1g22850) with the best hit found in yeast (Tvp38p) and in the Arabidopsis proteome (At2g02370). Identical residues are shown in black and conserved residues are shown in gray. Red color shows the vesicle associated protein domain.

Tvp38p       1 ---------------MSQSYEAGNANMGQ-GEDDEFDGYFEDFDND--------------
AT2G02370    1 -----------------------------------------------MSNPLKESR----
At1g22850    1 MRSLTLGLTLRSSSSSSLPFTCNSRFCSPPPPSSSFR-LYKRFHFLKPCSSLKQTKKKKQ


Tvp38p      31 -IMPNSNNGQRVGTNAGLSFNDEVNVNDDDFLDIYNMSPRERLMHNIRKNVQKLQFYFYS
AT2G02370   10 EDIANSTPHMR---DNEYV-RLVVAHE---------ASPAETVLSLSQSEVQSKKFM---
At1g22850   60 QSLPSTAPPPQ---SLRWFFNSKSTND---------ENDEDDVKSESDDDGGSEGD----


Tvp38p      90 LRLWQQIII-----VLLGIMLMIMGILLLVFHNAIL---HKVVVTSNDLREKMSTHFILM
AT2G02370   54 --WWLKALGICAVALLLTLVFGKWGVP-FVFQKVLIPILQW-E--ATAFGRPMLAIVLVV
At1g22850  104 --AAIK-GTILAGVLLIGTVGGFAGVG-YVYRDQINTFLTQFSTYIEGYGTAGYALFI-A


Tvp38p     142 VLIFFVAFPPMI-GYSLLSTTTGLIYGVSFEGWVTLALGSVTGSIASFVVFKTILHSRAE
AT2G02370  108 SL---ALFPVFLIPSGPSMWLAGMIFGYGL-GFVIIMVGTTIGMVLPYLIGLMFRD-RLH
At1g22850  159 VY---AGLEILAIPALPLTMSAGLLFGPLI-GTIIVSISGTMAASVAFLIARYFARERIL


Tvp38p     201 KLVHLNRRFEALASILQ---ENNSYWILALLRLCP-FPYSLTNGAIAGVYGISVRNFSI-
AT2G02370  163 QWLKRWPRQAAVLRLAAEGSWFHQFRVVAIFRVSP-FPYTIFNYAIVVT-SMRFWPYFFG
At1g22850  215 KLVEDNKKFLAI----DKAIGENGFRVVTLLRLSPLLPFSLGNYLYGLT-SVKFVPYVLG


Tvp38p     256 ANIITTPKLFIYLFIGSRVKSLAESEST-----GSRVFDLVSI---IITLLILSLTAWLL
AT2G02370  221 SIAGMIPEAFIYIYSGRLIRTFADVQY------GHQRLTTVEIVYNVISLVIAVVTTVAF
At1g22850  270 SWLGMLPGSWAYVSAGAFGRAIIQEESNVGLPGGNGQLLTLGV-----GLLVTALAGTYV


Tvp38p     308 YFKTKKRYLELQNRDRQV-STDQLPELSFEV---------------
AT2G02370  275 TVYAKRALRELQNAEANEDEEVQVRKVRFEMKNVVQHEEDNHQRLP
At1g22850  325 TSLAKDAIKDIDDDEKRDAK--------------------------
