# Supplementary material for: New Putative Chloroplast Vesicle Transport Components and Cargo Proteins Revealed Using a Bioinformatics Approach: An Arabidopsis Model
Source: PLoS One. 2013 Apr 1;8(4):e59898. doi: 10.1371/journal.pone.0059898 (PMC3613420; doi:10.1371/journal.pone.0059898)
Supplement: Figure S8 — A multiple sequence alignment of the putative chloroplast VAP protein (At4g05060) with the best hit found in yeast (Scs2p), human (VAPA) and the Arabidopsis proteome (At2g45140). (RTF) [file pone.0059898.s008.rtf]

Figure S8. A multiple sequence alignment of the putative chloroplast VAP protein (At4g05060) with the best hit found in yeast (Scs2p), human (VAPA) and the Arabidopsis proteome (At2g45140). Identical residues are shown in black and conserved residues are shown in gray. Red color shows the MSP domain.

Scs2p        1 ------------------------------------------------------------
VAP          1 ------------------------------------------------------------
At2g45140    1 ------------------------------------------------------------
At4g05060    1 MALTEDKSDSDGRRWGKFKLPFRNSNSQAPSASSSSSMATSSSSVTSSHLNQNYIHQSRH


Scs2p        1 -------------------------------------MSAVEISPDVLVYKSPLTEQSTE
VAP          1 -------------------------MASASGAMAKHEQILVLDPPTDLKFKGPFTDVVTT
At2g45140    1 -----------------------------------MSNELLTIDPVDLQFPFELKKQISC
At4g05060   61 FQYHGPPVVEGLGQNHHQSAATIPSMSSVARSLLPTKRRLKLDPSAKLYFPYEPGKQVRS


Scs2p       24 YASISNNSDQTIAFKVKTTAPKFYCVRPNAAVVAPGETIQVQVIFLGLTEEPAAD-----
VAP         36 NLKLRNPSDRKVCFKVKTTAPRRYCVRPNSGIIDPGSTVTVSVMLQPFDYDP-N------
At2g45140   26 SLYLGNKTDNYVAFKVKTTNPKKYCVRPNTGVVHPRSSSEVLVTMQAQKEAP-AD-----
At4g05060  121 AIKIKNTSKSHVAFKFQTTVPKSCFMRPAGAILAPGEEIIATVFK--FVEPPENNEKPME


Scs2p       79 FKCRDKFLVITLPSPYDLN-GKAVADVWSDLEAEFKQQAISKKIKVKYLISPDVHPAQNQ
VAP         89 EKSKHKFMVQTIFAPPNTSDMEA---VWKEAKPD---ELMDSKLRCVFEMPNENDKLGI-
At2g45140   80 LQCKDKFLLQCVVASPGATPKDVTHEMFSKEAGH---RVEETKLRVVYVAPPRPPSPVRE
At4g05060  179 QKSGVKFKIMSLKMK---VPTDYMPELFEEQKDH---VSEEQVMRVVFLDPENPNSMMEK


Scs2p      138 NIQ-----ENKETVEPVVQ-------------DSEPKEVPAVVNEK-EVPAEPETQPPVQ
VAP        142 -TP----PGNAPTVTSMSSINNTVATPASYHTKDDPRGLSVLKQEKQKNDMEPSKAVPLN
At2g45140  137 GSE----EGSSPRAS-VSDNG-------------------------NASDFTA--APRFS
At4g05060  233 LKSQLAEADAADEARKKASEG--IV-------GPKPIGEGLVIDEWKQ------RRERYL


Scs2p      179 VKKEEVP---------------PVVQKTVPHENEK-QTSNSTPAPQNQIKE---------
VAP        197 ASKQDGPMPKPHSVSLNDTETRKLM-----------EECKRLQGEMMKLSEENRHLRDEG
At2g45140  165 ADRVDA--------QDNSSEARALVTKLTEEKNSAVQLNNRLQQELDQLRRESKR-----
At4g05060  278 AQQQGGV---------------------------------------DAA-----------


Scs2p      214 ------------A--ATVPA-E---NESSSMGIFILVALLILVLGWFYR--
VAP        246 LRLRKVAHSDKPGSTSTASFRDNVTSPLPSLLVVIAAIFIGFFLGKFIL--
At2g45140  212 ---------SKS-------------GGIPFM-YVLLVGLIGLILGYIMKRT
At4g05060      ---------------------------------------------------
