# Supplementary material for: New Putative Chloroplast Vesicle Transport Components and Cargo Proteins Revealed Using a Bioinformatics Approach: An Arabidopsis Model
Source: PLoS One. 2013 Apr 1;8(4):e59898. doi: 10.1371/journal.pone.0059898 (PMC3613420; doi:10.1371/journal.pone.0059898)
Supplement: Figure S9 — A multiple sequence alignment of the putative chloroplast AtRabA5e protein (At1g05810) with the best hit found in yeast (YPT31p), and human (Rab11A). (RTF) [file pone.0059898.s009.rtf]

Figure S9. A multiple sequence alignment of the putative chloroplast AtRabA5e protein (At1g05810) with the best hit found in yeast (YPT31p) and human (Rab11A). Identical residues are shown in black and conserved residues are shown in gray. Red color shows the Rab domain.

Ypt31p       1 -------------------------------------------MSSEDYGYDYDLLFKIV
Rab-11A      1 -------------------------------------------MG--TRDDEYDYLFKVV
At1g05810    1 MSSCASLLHRLPSPPLSLSLSLQTSPTSLSRNLGKKKKTVKRAMSSDD-EGREEYLFKIV


Ypt31p      18 LIGDSGVGKSNLLSRFTKNEFNMDSKSTIGVEFATRTLEIDGKRIKAQIWDTAGQERYRA
Rab-11A     16 LIGDSGVGKSNLLSRFTRNEFNLESKSTIGVEFATRSIQVDGKTIKAQIWDTAGQERYRA
At1g05810   60 VIGDSAVGKSNLLSRYARNEFSANSKATIGVEFQTQSMEIEGKEVKAQIWDTAGQERFRA


Ypt31p      78 ITSAYYRGAVGALIVYDISKSSSYENCNHWLSELRENADDNVAVGLIGNKSDLAHLRAVP
Rab-11A     76 ITSAYYRGAVGALLVYDIAKHLTYENVERWLKELRDHADSNIVIMLVGNKSDLRHLRAVP
At1g05810  120 VTSAYYRGAVGALVVYDITRRTTFESVGRWLDELKIHSDTTVARMLVGNKCDLENIRAVS


Ypt31p     138 TEESKTFAQENQLLFTETSALNSENVDKAFEELINTIYQKVSKHQMDLGDSSANGNANGA
Rab-11A    136 TDEARAFAEKNGLSFIETSALDSTNVEAAFQTILTEIYRIVSQKQMSDRRENDM------
At1g05810  180 VEEGKALAEEEGLFFVETSALDSTNVKTAFEMVILDIYNNVSRKQLNSDTYKDELTVNR-


Ypt31p     198 SAPNGPTISLTPTPNENKKANGNNCC---
Rab-11A    190 -SPSNNVVPIHVPP-TTENKPKVQCCQNI
At1g05810  239 ------VSLVKDDNSASKQSSGFSCCSST
