# Supplementary material for: New Putative Chloroplast Vesicle Transport Components and Cargo Proteins Revealed Using a Bioinformatics Approach: An Arabidopsis Model
Source: PLoS One. 2013 Apr 1;8(4):e59898. doi: 10.1371/journal.pone.0059898 (PMC3613420; doi:10.1371/journal.pone.0059898)
Supplement: Figure S10 — A multiple sequence alignment of the putative chloroplast AtRabF1 protein (At3g54840) with the best hit found in yeast (Vps12p) and human (Rab5B). (RTF) [file pone.0059898.s010.rtf]

Figure S10. A multiple sequence alignment of the putative chloroplast AtRabF1 protein (At3g54840) with the best hit found in yeast (Vps12p) and human (Rab5B). Identical residues are shown in black and conserved residues are shown in gray. Red color shows the Rab domain.

Vps21p       1 --------------------------MNTSVTSIKLVLLGEAAVGKSSIVLRFVSNDFAE
Rab-5B       1 -------------MTSRSTARPNGQPQASKICQFKLVLLGESAVGKSSLVLRFVKGQFHE
At3g54840    1 MGCASSLPDRNSGTLSGLSNSENAVPADAKNLRVKLVLLGDSGVGKSCIVLRFVRGQFDA


Vps21p      35 NKEPTIGAAFLTQRVTINE-HTVKFEIWDTAGQERFASLAPMYYRNAQAALVVYDVTKPQ
Rab-5B      48 YQESTIGAAFLTQSVCLDD-TTVKFEIWDTAGQERYHSLAPMYYRGAQAAIVVYDITNQE
At3g54840   61 TSKVTVGASFLSQTIALQDSTTVKFEIWDTAGQERYSALAPLYYRGAGVAVIVYDITSPE


Vps21p      94 SFIKARHWVKELHEQASKDIIIALVGNKIDMLQEGGERKVAREEGEKLAEEKGLLFFETS
Rab-5B     107 TFARAKTWVKELQRQASPSIVIALAGNKADLAN---KRMVEYEEAQAYADDNSLLFMETS
At3g54840  121 SFKKAQYWVKELQKHGSPDIVMALVGNKADLHE---KREVPTEDGMELAEKNGMFFIETS


Vps21p     154 AKTGENVNDVFLGIGEKIPLKTAEEQNSASNERESNNQRVDLNAANDGTSANSACSC-
Rab-5B     164 AKTAMNVNDLFLAIAKKLPKSEPQNLGGAAGRS----RGVDLHEQS--QQNKSQCCSN
At3g54840  178 AKTADNINQLFEEIGKRLPRPAPSS---------------------------------
