# Supplementary material for: New Putative Chloroplast Vesicle Transport Components and Cargo Proteins Revealed Using a Bioinformatics Approach: An Arabidopsis Model
Source: PLoS One. 2013 Apr 1;8(4):e59898. doi: 10.1371/journal.pone.0059898 (PMC3613420; doi:10.1371/journal.pone.0059898)
Supplement: Figure S11 — A multiple sequence alignment of the putative chloroplast AtRabB1c protein (At4g35860) with the best hit found in yeast (YPT1p) and human (Rab2A). (RTF) [file pone.0059898.s011.rtf]

Figure S11. A multiple sequence alignment of the putative chloroplast AtRabB1c protein (At4g35860) with the best hit found in yeast (YPT1p) and human (Rab2A). Identical residues are shown in black and conserved residues are shown in gray. Red color shows the Rab domain.

Ypt1p        1 MNSEYDYLFKLLLIGNSGVGKSCLLLRFSDDTYTNDYISTIGVDFKIKTVELDGKTVKLQ
Rab-2A       1 --MAYAYLFKYIIIGDTGVGKSCLLLQFTDKRFQPVHDLTIGVEFGARMITIDGKQIKLQ
At4g35860    1 --MSYDYLFKYIIIGDTGVGKSCLLLQFTDKRFQPVHDLTIGVEFGARMVTVDGRPIKLQ


Ypt1p       61 IWDTAGQERFRTITSSYYRGSHGIIIVYDVTDQESFNGVKMWLQEIDRYATSTVLKLLVG
Rab-2A      59 IWDTAGQESFRSITRSYYRGAAGALLVYDITRRDTFNHLTTWLEDARQHSNSNMVIMLIG
At4g35860   59 IWDTAGQESFRSITRSYYRGAAGALLVYDITRRETFNHLASWLEDARQHANPNMSIMLIG


Ypt1p      121 NKCDLKDKRVVEYDVAKEFADANKMPFLETSALDSTNVEDAFLTMARQIKESMSQQNLNE
Rab-2A     119 NKSDLESRREVKKEEGEAFAREHGLIFMETSAKTASNVEEAFINTAKEIYEKIQEGVFDI
At4g35860  119 NKCDLAHKRAVSKEEGQQFAKEHGLLFLEASARTAQNVEEAFIETAAKILQNIQDGVFDV


Ypt1p      181 TTQK---KEDKG-----NVNLKGQSLTNTGGGCC-
Rab-2A     179 NNEANGIKIGPQHAATNATHAGNQGGQQAGGGCC-
At4g35860  179 SNESSGIKIGYGRTQ--GAAGGRDGTISQGGGCCG
