# Supplementary material for: New Putative Chloroplast Vesicle Transport Components and Cargo Proteins Revealed Using a Bioinformatics Approach: An Arabidopsis Model
Source: PLoS One. 2013 Apr 1;8(4):e59898. doi: 10.1371/journal.pone.0059898 (PMC3613420; doi:10.1371/journal.pone.0059898)
Supplement: Figure S12 — A multiple alignment of the putative chloroplast reticulon proteins (At2g20590, At4g28430, At5g58000) with the best hit found in yeast (RTN1) and the Arabidopsis proteome (At4g11220). (RTF) [file pone.0059898.s012.rtf]

Figure S12. A multiple alignment of the putative chloroplast reticulon proteins (At2g20590, At4g28430, At5g58000) with the best hit found in yeast (RTN1) and the Arabidopsis proteome (At4g11220). Identical residues are shown in black and conserved residues are shown in gray. Red color shows the reticulon domain.
RTN1_YEAST    1 ------------------------------------------------------------
At4g11220     1 ------------------------------------------------------------
At2g20590     1 --MESTPPYHRSNTKSASRLQDSSNP----PNLSLDLVLSSP------------------
At4g28430     1 MDSTTTPPSLRSNTRSALRLARNNKTLVKSHIPSLDLVLLSPKNN--NGT---PYPS---
At5g58000     1 ----MTPRRSLSSS-------DSN---D--KSPSVSVVAKKARSESVEGIEKKTTPGRVK


RTN1_YEAST    1 ------------------------------------------------------------
At4g11220     1 ------------------------------------------------------------
At2g20590    37 ----------NTPNPSSPVPLRDILLLPPSPLRKSRTRLSDRLEM-------------TS
At4g28430    53 --------PVSLSSPSSPVTLREILLLSPSPLRKSRTRLSNRFDM-------------EA
At5g58000    45 KIRSEVCTTIVKAGEFDSVALRKVNSLPSPNSEKSDTKTEQEVTIIENSKIPEEVKEFGV


RTN1_YEAST    1 ------------------------------------------------------------
At4g11220     1 ----------------------MADEHKHEE-------------------SSPNLDPAVE
At2g20590    74 EDAMAVVRKRGK---------GKGGQKSLLASP--RNPRRSRRRSEAVEEKE----ANLV
At4g28430    92 AEAA-VTARRSK---------TKGGQNGLLASPSPRNFRRSRLRSEAMVDTKENTEPIVV
At5g58000   105 CQEMIVSAKSNENEQIDNGDQEIGDQDDYEEDGDEEEEREVEKKSVDVKEINVAKENRVG


RTN1_YEAST    1 -----------------------------------------MSA------------SAQH
At4g11220    20 VVERESLMEKLSEKIHHKGDSSSSSSSDDE------NEKKSSSSSPKSLKSKVYRLFG-R
At2g20590   119 IEEIV----KLPPRKR---KTNGRPKKDKQSSAPPLCS----SSDLPNTC----------
At4g28430   142 VTDEK----KQNQRKQ---KKLGRSKKEKHSSVPLLASPSPSSDQPQDVC----------
At5g58000   165 GVEIK----KFSQFQN---RTSPSPSSVRKISPPVIKRATSVYSAPPNSTSSTDRFAEQE


RTN1_YEAST    8 SQAQQQQQQKSCNCDLLLWRNPVQTGKYFGGSLLALLILKKV-NL--ITFFLKVAYTILF
At4g11220    73 ERPVHKVLGGGKPADIFMWKDKKMSGGVFGGATVAWVLFELM----EYHLLTLLCHVMIV
At2g20590   158 --QSDLNLIGEIISDLVMWRDVAKSTLWFGFGCLSFLSSCFA-KGVNFSVFSAVSNLGLV
At4g28430   185 --QGDLERIRENISDLIMWRDVAKSTLWFGFGCICFLSTCFAAKGFNFSVFSAISYLGLL
At5g58000   218 DNFTHSQSKLQSLVDLVMWRDVSRSTLVFGFGTFLIISSSYA-NDLNFSFISVVAYMGLI


RTN1_YEAST   65 TTGSIEFVSKLFLGQGLITKY------------GPKECPNIAGFIKPHIDEALKQLPVFQ
At4g11220   129 ------ALAVLFLWSNATMFIHKSPPKIPEVHIPEEPLLQLASGLRIEINRGI-------
At2g20590   215 ------LLCGSFLSNTLCQRK--NEDTKRAFHVSEDDVLRSARRVLPATNFFI-------
At4g28430   243 ------FLGVSFLSNTLRQRV--TEEARRELKLSEDDVLRIARRMLPITNLAI-------
At5g58000   277 ------YLGLMFVLKSLIHRGMVEEERHKVVGVREEDVKRMLRLIMPYLNESL-------


RTN1_YEAST  113 AHIRKTVFAQVPKHTFKT-AVALFLLHKFFSWFSIWTIVFVADIFTFTLPVIYHSYKHEI
At4g11220   176 -SSLREIASGRDIKKFLSAIAGLWVLSILGGCYSFLTLAYIALVLLFTVPLFYDKYEDKV
At2g20590   260 -SKTSELFSGEPSMTLKV-TPFLLLGAEYGHLITLWRLSAFGFFLSFTIPKLYSCYTHQI
At4g28430   288 -SKTSELFSGEPAMTLKV-APFVLMGAEYGYLITLWRLCAFGFFLSFTIPKLYSCYASQL
At5g58000   324 -HQLRALFSGDPSTTLKM-GVVLFVLARCGSSITLWNLAKFGFLGAFTIPKIFISYSTHF


RTN1_YEAST  172 DATVAQGVEISKQKTQEFSQMACEKTKPYLDKVESKLGPISNLVKSKTAPVSST------
At4g11220   235 DSYGEKAMAELKKQ---Y--AVLDAKVFS----KIPRGPLKD------------------
At2g20590   318 SQKVERVKTRIGEA---W--GVCSHKKIL----AGSAVTAFWNLTSIRTRIFAVFIILVI
At4g28430   346 NQKVECAQRRFVEA---W--GVCTHKKFV----AGSAVTAFWNLTSLKTRFIAVFIIVVV
At5g58000   382 SAYGNFWMRRFRDA---W--ESCNHKKAV----ALALFTLVWNLSSVTARVWAAFMLLVA


RTN1_YEAST  226 --AGPQ-TASTSKLAADVPLEPESKAYTSSAQVMPE----VPQHE----PSTTQEFNVDE
At4g11220   268 -----------------KKKD---------------------------------------
At2g20590   369 FRYRRQNLQLTPEEVEPVENEQEEETLPQEEETVPQEEETVPQEEEQTQPSEERALVV--
At4g28430   397 IRYRRQNLQLDSEDEEEKKQ--QEKTHPE-QQKSPEDKSTSPRS------AEEEQALV--
At5g58000   433 FRYYQHKMIWTTDQADDDEDDNEEEEAEEEKEQVPPKHKRAPPHMMM--PNKLKKIS---


RTN1_YEAST  275 LSNELKKSTKNLQNELEKNNA
At4g11220       ---------------------
At2g20590   427 VVAET----------------
At4g28430   446 LVAETKA-PKKLY--------
At5g58000       ---------------------
